# Moth declines are most severe in broadleaf woodlands despite a net gain in habitat availability

**DOI:** 10.1111/icad.12578

**Published:** 2022-04-28

**Authors:** Dan Blumgart, Marc S. Botham, Rosa Menéndez, James R. Bell

**Affiliations:** ^1^ Rothamsted Insect Survey, Biointeractions and Crop Protection Rothamsted Research West Common, Harpenden UK; ^2^ Centre for Ecology & Hydrology Crowmarsh Gifford, Wallingford, Oxfordshire UK; ^3^ Lancaster Environment Centre Lancaster University Lancaster UK

**Keywords:** broadleaf woodland, insect conservation, insect decline, traits based analysis, UK moth decline

## Abstract

1. While agricultural intensification and habitat loss are cited as key drivers of moth decline, these alone cannot explain declines observed in UK woodlands – a habitat that has expanded in area since 1968.
2. We quantified how moth communities changed across habitats and regions and determined how species traits interacted with habitat in predicting moth abundance change. We hypothesised that, in woodlands, species more vulnerable to shading and browsing by deer (species specialising on forbs, shrubs and shade‐intolerant plants) had declined more severely than other species, and that moth decline in woodlands was more severe at sites more susceptible to deer damage.
3. We modelled abundance, biomass, species richness and diversity from 1968 to 2016 and explored how these interacted with habitat and region. We also modelled the interaction between habitat and two moth species traits: larval feeding guild and shade‐tolerance of hostplant.
4. Moth declines were consistently highest in broadleaf woodland. Abundance, biomass, species richness and diversity declined significantly by −51%, −52%, −14% and −15% in woodlands, respectively, compared to national trends of −34%, −39%, −1% (non‐significant) and +10%. Declines were no greater in woodlands more susceptible to deer browsing damage. Traits based analysis found no evidence that shading and intensive browsing by deer explained moth declines in woodland.
5. Moth decline was more severe in broadleaf woodlands than in intensively managed farmlands. We found no evidence that deer browsing or increased shading has driven these trends: the primary cause of the decline of moths in woodlands remains unclear.

## Graphical abstract

- The abundance, biomass, species richness and diversity of moths in the UK was modelled from 1968 to 2016 across seven habitat types.
- Declines were most severe in broadleaf woodland and this was the only habitat in which declines in richness and diversity occurred. At the national level, diversity increased over this period.
- Traits based analysis of species trends provided no evidence that increased shadiness and overgrazing by deer were responsible for the decline of moths in woodland.

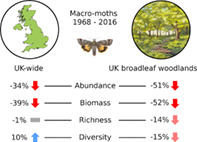

## INTRODUCTION

Changes to insect abundance and diversity have received much attention lately, with many studies reporting declines at both local and national scales in recent decades (Leather, 2017; Wagner, 2020), although some studies have found overall stability or increase in some groups and regions (Crossley et al., 2020; van Klink et al., 2020). Key drivers of insect decline are thought to be habitat loss, degradation and agricultural intensification (Fox, 2013; Wagner et al., 2021), with growing recognition of the role of light pollution in nocturnally active insects (Owens et al., 2019). In addition, the effects of recent climate change are complex and vary spatially and across species (Menéndez, 2007; Wilson & Fox, 2020), with positive (Fox et al., 2014; Warren et al., 2001), negative (Halsch et al., 2021; Salcido et al., 2020) and in some cases catastrophic (Janzen & Hallwachs, 2021) effects reported. The relative contribution of each of these drivers to the observed trends remains difficult to quantify, not least because many of the drivers are both interacting and co‐occurring (Wagner et al., 2021). While biodiversity loss in intensifying landscapes is unsurprising, more disturbing findings show insect declines in remote areas, far from direct human influence (Harris et al., 2019; Janzen & Hallwachs, 2019), suggesting a more insidious driver of decline, usually attributed to climate change.

Lepidoptera are one of the few insect groups that have been monitored systematically over a long time‐period and widespread declines across northern Europe have been reported in both moths (Antão et al., 2020; Conrad et al., 2006; Franzén & Johannesson, 2007; Groenendijk & Ellis, 2011; Mattila et al., 2006; Roth et al., 2021) and butterflies (Brereton et al., 2011; Maes et al., 2001; van Dyck et al., 2009; Wenzel et al., 2006). Trait‐based analyses of these groups have offered insights into the drivers of decline. Notably, the decline in butterflies that are specialists of dry, open, low‐fertility habitats across Europe and North America has been widely demonstrated (Habel et al., 2019; Pöyry et al., 2017; Wenzel et al., 2006) and has been attributed to nutrient enrichment through agricultural inputs and air pollution as well as direct habitat loss. Similar patterns have been observed in moths (Fox et al., 2014; Valtonen et al., 2017). In the United Kingdom, decline in moth abundance has been more severe in the south (Conrad et al., 2006; Fox et al., 2021), which may be due to higher rates of habitat loss through agricultural intensification, especially the south‐east. Declines in the north may also have been compensated for by many species expanding their ranges northwards (Fox et al., 2021).

Agricultural intensification is often cited as a critical driver of insect decline (Fox, 2013; Fox et al., 2021; Wagner et al., 2021) and some studies comparing trends in agricultural and non‐agricultural settings have found evidence to support this (Forister et al., 2010; Seibold et al., 2019). However, data on British moths suggest that declines in abundance and biomass since the late 1960s have been more severe in woodlands than in farmland (Bell et al., 2020; Macgregor et al., 2019) despite a net gain in extent of broadleaf woodland over the same time period; this finding warrants further investigation and is the topic of this article.

Moth declines in most UK habitat types are understandable due to the drivers listed above: agricultural intensification, habitat loss and light pollution. However, moth decline in broadleaf woodland is less easy to explain as this semi‐natural habitat type is presumably less susceptible to these drivers. Broadleaf woodland typically receives no direct inputs of synthetic pesticides or fertilisers and, unlike most UK semi‐natural habitats, it has expanded in area since the early 20th century: broadleaf woodland cover rose from 728,000 ha in 1967 (Hopkins & Kirby, 2007) to 1,573,000 in 2020 (Reid et al., 2021). Insects living in broadleaf woodland may also be more buffered against the direct effects of climate change such as extreme heat and drought (De Frenne et al., 2013). The lack of clear external drivers of decline points to a deterioration of habitat quality within the woodlands – a phenomenon that has been widely noted (Reid et al., 2021). Declines in abundance within woodlands have been recorded in butterflies (Fox et al., 2015), thought to be driven largely by canopy closure and shading (Fartmann et al., 2013; Sparks et al., 1996) due to the cessation of active woodland management (Kirby et al., 2017). However, being largely nocturnal, moths are less dependent on exposure to sunlight for successful breeding (Clench, 1966) and, unlike butterflies, the abundance and diversity of moths are often quite high in dense woodland interiors (Slade et al., 2013). The decline of woodland birds in the United Kingdom has been linked to the rapid increase in deer densities since the mid‐20th century resulting in damage to woodland understories and loss of ground flora (Fuller et al., 2005; Newson et al., 2012; Perrins & Overall, 2001). Deer browsing has been shown to negatively affect the abundance of Lepidoptera larvae (Baines et al., 1994) as well as other invertebrate groups in woodlands (Stewart, 2001), but this has not been linked to long‐term declines.

The Rothamsted Insect Survey (RIS) moth dataset has been used previously to determine which species traits are most common among species undergoing decline (Conrad et al., 2004; Coulthard et al., 2019) and to model the rates of decline across geographical regions (Conrad et al., 2006; Fox et al., 2013). However, rates of abundance and biomass change according to habitat types are only coarsely estimated (Bell et al., 2020; Macgregor et al., 2019). Furthermore, it is still not known whether UK moth species richness and diversity at the site‐level has changed. With range expansions in many species (Outhwaite et al., 2020; Randle, 2019) despite declines in abundance, species richness and diversity may have increased simultaneously, as is the case with moths in Finland (Antão et al., 2020) and butterflies in the United Kingdom (Menéndez et al., 2006). Here, we have addressed these knowledge gaps by modelling the response of four community attributes: abundance, biomass, species richness and diversity across seven habitat types (arable, broadleaf woodland, conifer plantation, improved grassland, other semi‐natural, upland and urban) and two regions (north and south) to produce percentage changes for each response. This is the first time for which changes in species richness and diversity has been modelled for UK moths. The biomass models allow for comparison with Macgregor et al. (2019) who claims there has been no decline in moth biomass – although using a potentially flawed model that we return to in the discussion. In addition, we tested whether key species traits interact with habitat in determining species trends. We focus on broadleaf woodlands as this habitat type is well sampled and the drivers of decline in this habitat type are largely unknown for moths. We include six other habitat types to act as a ‘control’ to compare against broadleaf woodland and we included region (north and south) as this is known to be an important predictor, with declines more severe in the south (Fox et al., 2021).

We hypothesised that moths that feed on plants vulnerable to increased shading and browsing by deer (forbs, shrubs and shade‐intolerant plants) have declined more in broadleaf woodlands than species that feed on plants less vulnerable to both shading and browsing (grasses, trees above the browse‐line, lichens and shade‐tolerant plants) (Gill & Beardall, 2001; Kirby, 2001; Morecroft et al., 2001). We predicted that this pattern would not be found in the other habitat types for which shading and browsing are not expected drivers of decline. The impact of deer across the country varies according to location and surrounding landscape (Spake et al., 2020). We predicted that declines in the four community attributes would be more severe in broadleaf woodlands in which the likelihood of deer browsing damage was higher.

## METHODS

### Moth data

Macro‐moth records from 1968 to 2016 were extracted from the Rothamsted Insect Survey (RIS) database for every site in the United Kingdom plus the Isle of Man. The RIS network consists of standardised light traps that operate every night of the year. The design, described by Williams (1948), has remained unchanged since the inception of the network. Moths are captured every night and are either identified daily or combined into multi‐day catches, depending on trap operator. Only sites that ran for at least 3 years between 1968 and 2016 were used in the analysis. Although 3 years is not long enough for a meaningful abundance trend at a single site, many short‐running sites can still contribute to multi‐site models for different regions and at a national scale, as has been done before in previous analysis of RIS data (Conrad et al., 2004; Fox et al., 2021; Harrower et al., 2019).

### Land‐use data and habitat allocation

Land‐use data (25 m raster) were extracted from the Land Cover Map 2015 (LCM 2015) for Great Britain (Rowland et al., 2017a) and Northern Ireland (Rowland et al., 2017b). Using ArcMap version 10.4 (ESRI, 2018), buffers of 500 m radii were drawn around each site and the area of each land‐use type within each buffer was calculated. This spatial scale was chosen as it was large enough to adequately capture the dominant surrounding land‐use type but still reflected the land‐use directly around the trap. When larger buffers were attempted, we found that either improved grassland or arable land came to dominate the circle in most cases, reducing the sample size of sites in other categories to the point where they could not be meaningfully analysed. The attractive radius of a moth trap, while not yet measured specifically for Rothamsted traps, is thought to be in the region of 30 m (Merckx & Slade, 2014; van Grunsven et al., 2014). Furthermore, the effect of surrounding habitat on local moth abundance and richness tends to be most important at radii below 500 m (Fuentes‐Montemayor et al., 2011; Woiwod & Gould, 2008).

Sites were categorised into seven habitat types based on their dominant land‐use type: arable, broadleaf woodland, conifer plantation, improved grassland, ‘other semi‐natural’, upland, and urban (Figure S1). These seven habitat types were chosen as they represent key land‐use types in the United Kingdom. Due to low sample size of upland sites (300 m or above), these could not be further divided into more specific land uses. All other habitat types are lowland sites (below 300 m). The habitat type at ‘other semi‐natural’ sites are all open, typically low‐nutrient environments that serve as a contrast against the other habitats. To avoid ambiguity, this habitat type is always written in inverted commas when referred to in the text. To examine the effect of latitude, the UK was then split into two regions: north and south at the 4500 N gridline on the British National Grid (≈53.9° latitude), following Conrad et al. (2006). Table S1 shows the distribution of sites across the seven habitat types and two regions. For each broadleaf woodland site, a deer damage estimator was calculated using the online Deer Damage Tool (Spake et al., 2020). This tool was developed using data from Britain's National Forest Inventory, which comprises over 15,000 forest plots. For each broadleaf woodland site, we provided the tool with the following site attributes: forest type, landscape forest cover, perennial cover, road density and grid square. The first three of these were calculated using the LCM2015. Road density was measured in ArcMap using data from OS OpenRoads (https://www.ordnancesurvey.co.uk/business-and-government/products/os-open-roads.html). Three required variables were unknown so were set to their mid‐values: tree density, tree volume and tree age range. Definitions of each of these terms can be found in the study by Spake et al. (2020). The tool provides a deer damage probability (between 0 and 1) for each site. This value was taken as an estimate of the likelihood of deer browsing damage at each broadleaf woodland site. Some sites in Wales could not be estimated as these fell outside of the area included in the online tool – these sites were excluded from this part of the analysis.

### Species traits data

Two species traits were chosen: feeding guild and the Ellenberg value for light‐affinity of the larval hostplant. These two traits are known to be important predictors of moth trends in abundance and occupancy (Conrad et al., 2004; Coulthard et al., 2019; Fox et al., 2014). Species traits were extracted from Waring and Townsend (2017) and Sterling and Henwood (2020) and Ellenberg numbers were extracted from Hill et al. (1999). Moths were split into eight feeding guilds: conifers, broadleaf shrubs, broadleaf trees, broadleaf polyphagous, forbs, grasses, highly polyphagous and lichens. Species that feed primarily on moss, detritus or stored goods were excluded because they were too few. Table S2 describes the levels in the feeding guild trait, including a description of which plant species were defined as ‘trees’ or ‘shrubs’. Ellenberg values were only used for moth species with three or fewer hostplants. When species had either two or three hostplants, the mean Ellenberg number of the hostplants was used.

### Analysis

Analysis was carried out in R version 4.0.3 (R Core Team, 2020) and was split into two parts: (1) ‘general’ analysis and (2) ‘species‐specific’ analysis. General analysis combined all moth counts in a single site‐year into four response variables: total abundance, biomass, species richness and diversity. The species‐specific analysis considered the abundance trend of each individual species and allows species‐specific traits to be used as fixed effects in the models. All analysis can be replicated using the R scripts provided in the Supporting Information S1.

#### General analysis: Total abundance, biomass, species richness and diversity

For these analyses, only ‘complete’ site‐years were used, as defined by Conrad et al. (2004): no gaps in recording of more than 2 weeks from 1 April to 31 October or more than 4 weeks from 1 November to 31 March. This narrowed the dataset to 266 sites. Four response variables were calculated: (1) total abundance, (2) biomass, (3) species richness and (4) species diversity. Total abundance was the sum of all moths caught in one site‐year. For biomass, dry weight estimates from Kinsella et al. (2020) were used and summed per site‐year. This technique estimates the mean biomass of each moth based on its family and wingspan. For aggregate species such as *Oligia latruncula/strigilis/versicolor*, a biomass estimate was calculated by taking the mean average of all the species in the group. Species richness (Hill number 0) was estimated asymptotically using the iNEXT package (Hsieh et al., 2016) and was expressed as the estimated number of species per site‐year. Species diversity was also estimated asymptotically in the iNEXT package using Hill number 1 – the exponential of the Shannon diversity index. This was expressed as the ‘effective number of common species’; see Chao et al. (2014) for more details on Hill numbers.

For each response variable, four models were run using both a linear and non‐linear technique, resulting in eight models per response variable. Models were run to assess the effect (1) year, (2) year interacting with region, (3) year interacting with habitat and (4) year interacting with region within broadleaf woodland only. A three‐way interaction between year, region and habitat was attempted, but this was not possible due to sparseness of sites, especially in the north, so the effect of region within broadleaf woodland only was used instead. The linear models were used to assess the direction, magnitude and significance of trends, while non‐linear models were constructed primarily for visualisation and to detect any non‐linear changes in the response variables over time. Non‐linear models were considered a better fit than linear models if their AICc value was at least two values lower than that of the linear model.

For each model, a generalised additive mixed model (GAMM) was fit using the mgcv package (Wood, 2017). In the first case, each response variable was modelled as a function of year (as a continuous variable), with a random intercept for year (as a factor), a random intercept for site and a random slope for year within each site. The random effects structure was coded as “s(Year_factor, bs = “re”) + s(Year, Site_factor, bs = “re”) within the ‘gam’ function. In linear models, the fixed effect year was included as a parametric term, and in non‐linear models, year was included as a smooth term using thin plate regression splines. The second model introduced an interaction with region (factor with two levels: north/south). In the linear model, this was included as a parametric term. In the non‐linear model, a smooth for the effect of year in each region was estimated, plus a parametric term for region that allowed the intercept to vary. The third model was structured the same way as the second, but with habitat (factor with seven levels) replacing region. In the fourth model, only data from broadleaf woodland sites were used, and an interaction between year and region was modelled as above on this subset. A negative binomial error distribution was used for abundance models. A Gaussian error distribution was used for biomass (log‐transformed), species richness and diversity models.

In linear models, the statistical significance of year and its interaction with region or habitat was assessed using the anova.gam function in mgcv. This is the equivalent of a type III ANOVA, where one term is removed from the model and the full model is compared to the reduced model. In non‐linear models, a reduced model was run with the interaction term omitted and was compared to the full model using the anova.gam function. Model terms were considered significant if *p* < 0.05.

If an interaction was significant, a post hoc analysis was carried out to determine which of the trends differed significantly from zero. The emtrends function in the emmeans package (Lenth, 2019) was used to estimate the marginal mean year coefficients for each level within region/habitat along with 95% confidence intervals (CI). Trends were considered significant if the 95% CI did not include zero. Finally, percentage change was estimated for each response variable using the model predictions (on the response scale) for the first and final years in the time series.

Sensitivity analysis was carried out in each model using a jackknife method. One site at a time was excluded from the data and the model was rerun. Year coefficients and percentage change from the jackknifed models were compared to the full models to determine whether any individual sites were having an overwhelming influence on the results. In addition, the package poptrends (Knape, 2016) was used to model total abundance as a function of year in order to test for significant periods of decline/increase within the time series. In these models, random effects were structured the same as in the GAMMs above. As this package cannot handle interaction effects, a separate model was run for each factor level. The number of knots (k) was started at 16 and generalised cross‐validation in the underlying mgcv package was used to find the optimal ‘wiggliness’ of the trend.

The effect of deer damage was modelled for broadleaf woodland sites only. One model was run for each of the four response variables. The models were structured in the same way as the linear models described above but only contained two parametric terms: year and deer damage estimate, and an interaction between the two. The significance of the year and deer damage interaction was assessed with the gam.anova function.

#### Species‐specific analyses

In order to maximise the amount of data available, incomplete site‐years were used for individual species models. The missing counts were estimated for each species across the specific species flight period using GAM following (Harrower et al., 2019). See Supporting Information S1 (estimating site‐year completeness) for a full description. This process also generated an estimate of the completeness of sampling for each species‐site‐year. In all models, to prevent spurious estimations, any species‐site‐year combination with a completeness of less than 0.5 was omitted.

##### Species‐specific trends and interactions with habitat and region

For each of 427 species, the change in estimated annual abundance was modelled using the poptrend package as above. As before, only sites with at least 3 years' worth of data were used (only including years with a completeness score of at least 0.5), resulting in 384 sites used in this part of the analysis. Twelve abundance trend estimates were modelled for each species: one model included all sites, and the remaining 11 included only subsets of sites. Trends were modelled in each region (two models) and in each habitat (seven models) and in each region within broadleaf woodland only (two models). Models were excluded if they were not of sufficient quality: that is, they did not contain enough sites, site‐years or individual moths (see Supporting Information S1: Model quality‐control). The cut‐off points were arrived at through trial‐and‐error and cut out most of the dubious model estimates as well as model convergence failures that would have otherwise occurred. The quality‐control step only applied to species‐specific trends and not to the ‘general’ analysis. The estimated percentage change for each species‐habitat/species‐region combination was stored and these were used as the response variable in the next stage of modelling. In four species, there were cases where no moths were present in the first half of the time series, meaning that the estimated percentage could not be meaningfully estimated and was extremely high. In these cases, the trend was remodelled starting at halfway through the time series in the year 1993.

The effect of habitat and region on species‐specific trends were assessed with linear mixed‐effects models (LMM) in the lme4 package (Bates et al., 2015) using the lmer function. The trends of each species within each subset were used as the response variable. The trends were ln(x + 100) transformed following Dennis et al. (2019) so that the distribution of trends approximated a normal distribution. Three models were run: trend was modelled as a function of (1) habitat, (2) region, and (3) region within broadleaf woodlands, with a random intercept for each species. Each observation was weighted according to its log‐transformed total sample size (i.e. the total number of individual moths of each species caught in each subset) using the ‘weights = ‘argument. As the uncertainty of the trend was greater for trends with smaller sample sizes, this ensured that more weight was given to trends with more certainty. To test whether the interaction was significant, a reduced model with the interaction term omitted was compared against the full model with a likelihood ratio test (LRT). The contrasts in the estimated marginal mean trend between levels of habitat/region were calculated using the emmeans() function with the Tukey method for multiple comparisons. For each habitat/region, 95% CIs for estimated marginal mean trend were produced. Trends were considered significant if the CIs did not include zero.

##### Species traits and interactions with habitat and region

Two traits were modelled: feeding guild (factor with eight levels) and Ellenberg value for light (continuous). As Ellenberg value for light was only applicable to a smaller subset of species (105/427), the two traits were modelled separately so that the feeding guild model could use all the available species. Three models were run for each trait. Trend (ln(x + 100)‐transformed) was modelled as a function of the trait interacting with (1) habitat, (2) region or (3) region within broadleaf woodland. As before, the lmer function was used, with a random intercept for species and each observation weighted by the log of its sample size. The significance of the interaction effect was assessed by dropping the interaction from the model and comparing the reduced model against the full model with an LRT as before. For continuous trait variables, post hoc tests were performed using the emtrends function to determine which slopes differed significantly from zero. The 95% CIs of the estimated marginal means of the slopes were also extracted using this function – they were considered significant if the CIs did not overlap zero. In addition to the trait interaction models, the effect of each trait was modelled alone, without any habitat/region term, to examine the overall effect of traits on species trends. The overall abundance trend for each species was used as a response variable. In these cases, a simple linear model with no random effects was run, again, using log‐sample size as a weighting factor for each species. The significance of the species trait in predicting trend was tested by running a reduced model and comparing it to the full model with an *F*‐test.

## RESULTS

### General analysis: Total abundance, biomass, species richness and diversity

**FIGURE 1:**
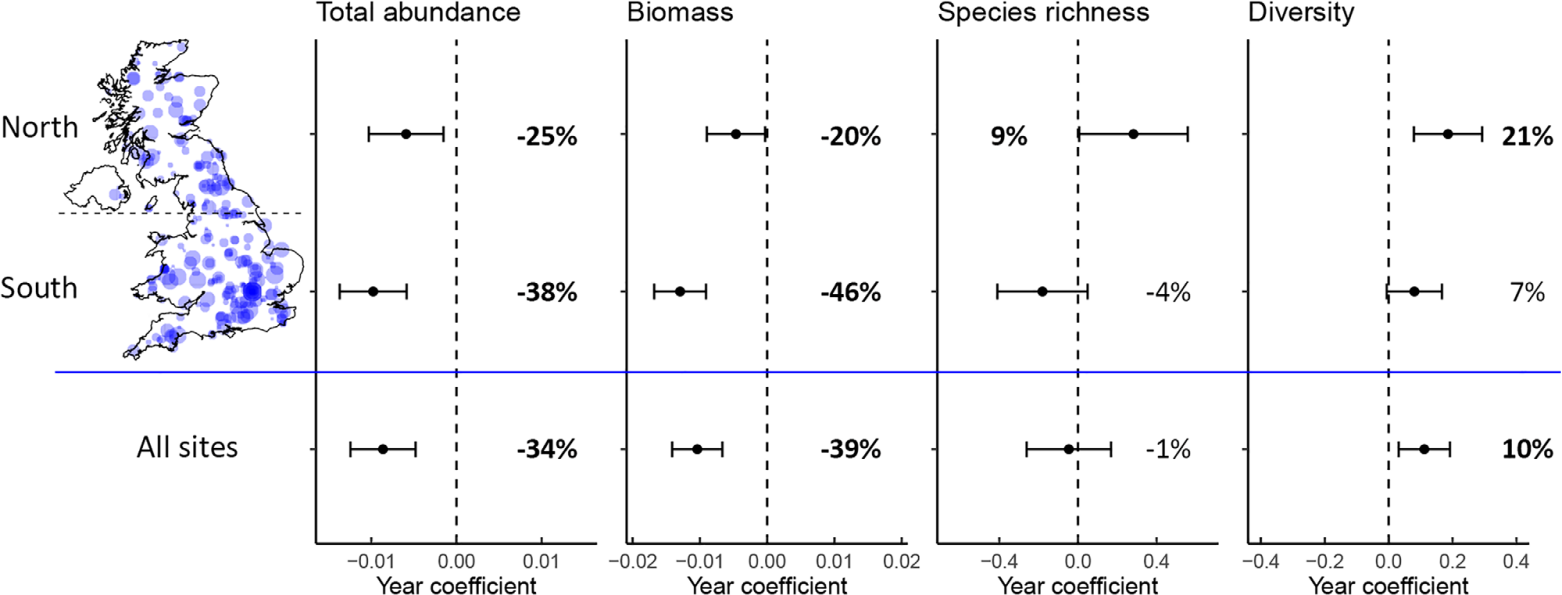
Year coefficients from the parametric components of GAMMs with 95% confidence intervals for north, south and all UK sites. Percentage change over 1968–2016 is shown next to the error bars with significant trends in bold. Map displays the location of sites used in the models with size of points proportional to the time‐series length of the trap. Dashed lines show no change (i.e. zero coefficient). Note that coefficients are for different measures of moth community attributes

A total of 8,829,484 moths were recorded across 266 sites, 49 years, and 3055 site‐years. Total moth abundance significantly declined by 34%, biomass significantly declined by 39%, species richness showed no significant directional change and species diversity significantly increased by 10% (Figure 1 and Table 1). For species richness, a non‐linear model performed better than a linear model (ΔAICc = 3.9) and showed a significant non‐linear trend with a small increase in richness up to the year 1990 followed by a decline to former levels (Figure S2c).

**TABLE 1:** Model AICc and *p* values used to determine the significance of the effect of year and the interaction between year and region/habitat

Explanatory variable(s)	Response variable	Linear/non‐linear	AICc	*p*
Year	Total abundance	Linear	48541.55	<0.0001
	Biomass	Linear	2335.06	<0.0001
	Species richness	Non‐linear	28869.86	0.00064
		Linear	28873.81	0.67
	Diversity	Linear	23449.32	0.0064
Year * region	Total abundance	Linear	48537.04	0.015
	Biomass	Linear	2306.40	<0.0001
	Species richness	Linear	28852.79	0.00026
	Diversity	Non‐linear	23430.11	0.00023
		Linear	23444.05	0.042
Year * habitat	Total abundance	Non‐linear	48458.11	<0.0001
		Linear	48512.61	<0.0001
	Biomass	Non‐linear	2273.87	<0.0001
		Linear	2329.44	0.0096
	Species richness	Non‐linear	28846.92	0.00027
		Linear	28853.66	<0.0001
	Diversity	Non‐linear	23397.53	<0.0001
		Linear	23401.16	<0.0001
Year * region (broadleaf woodland sites only)	Total abundance	Non‐linear	9258.684	<0.0001
		Linear	9273.784	0.0017
	Biomass	Non‐linear	143.8704	<0.0001
		Linear	160.2371	<0.0001
	Species richness	Non‐linear	5349.431	0.11
		Linear	5358.454	0.35
	Diversity	Non‐linear	4465.368	<0.0001
		Linear	4482.204	0.021

*Note*: Where non‐linear models were a superior fit to linear models (ΔAICc > 2) then the non‐linear models are presented as well. Asterisks denote the significance of year effect or the interaction effect between year and habitat/region (*p* >= 0.05 ‘ns’; <0.05 ‘*’; <0.01 ‘**’; < 0.001 ‘***’). AICcs are shown to compare the linear to the non‐linear version of the same model and can also be used to compare models with the same response variable and the same data.

#### Effect of region

There was a significant interaction effect between year and region for all four response variables (Figure 1 and Table 1). In each case, trends in the south were more negative than those in the north. For diversity, a non‐linear model outperformed a linear model (ΔAICc = 13.9) with both north and south showing an increasing trend until roughly 1990, after which they plateaued, although the trend in the south was more variable over time (Figure S3d).

#### Effect of habitat

**FIGURE 2:**
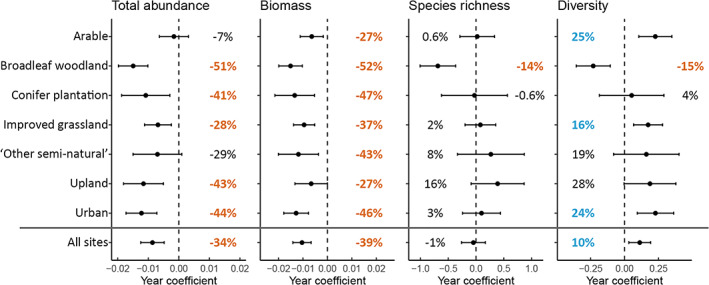
Year coefficients from the parametric components of GAMMs with 95% confidence intervals for four response variables in seven habitat types and for all sites combined. Percentage change over 1968–2016 is shown next to the error bars with significant trends in bold. Negative trends are coloured orange and positive trends blue. Dashed lines show no change (i.e. zero coefficient). Note that coefficients are for different measures of moth community attributes

There was a significant interaction between year and habitat for each of the four response variables (Figure 2 and Table 1). Broadleaf woodland was the only habitat in which all four response variables declined significantly. This habitat type showed the most severe declines out of any habitat type for all response variables: abundance −51%, biomass −52%, species richness −14% and diversity −15%. Species diversity was the only response variable in which significant positive trends occurred: for arable, improved grassland and urban sites. Abundance declined significantly in five out of the seven habitat types, with no significant trend in either arable or ‘other semi‐natural’. Biomass declined significantly in all habitat types. In all four response variables, a non‐linear model performed better than a linear model (Figure S4). Poptrend analysis showed that abundance in broadleaf woodland was stable up to the late‐1980s after which it declined severely (Figure S5).

#### Effect of region within broadleaf woodland

**FIGURE 3:**
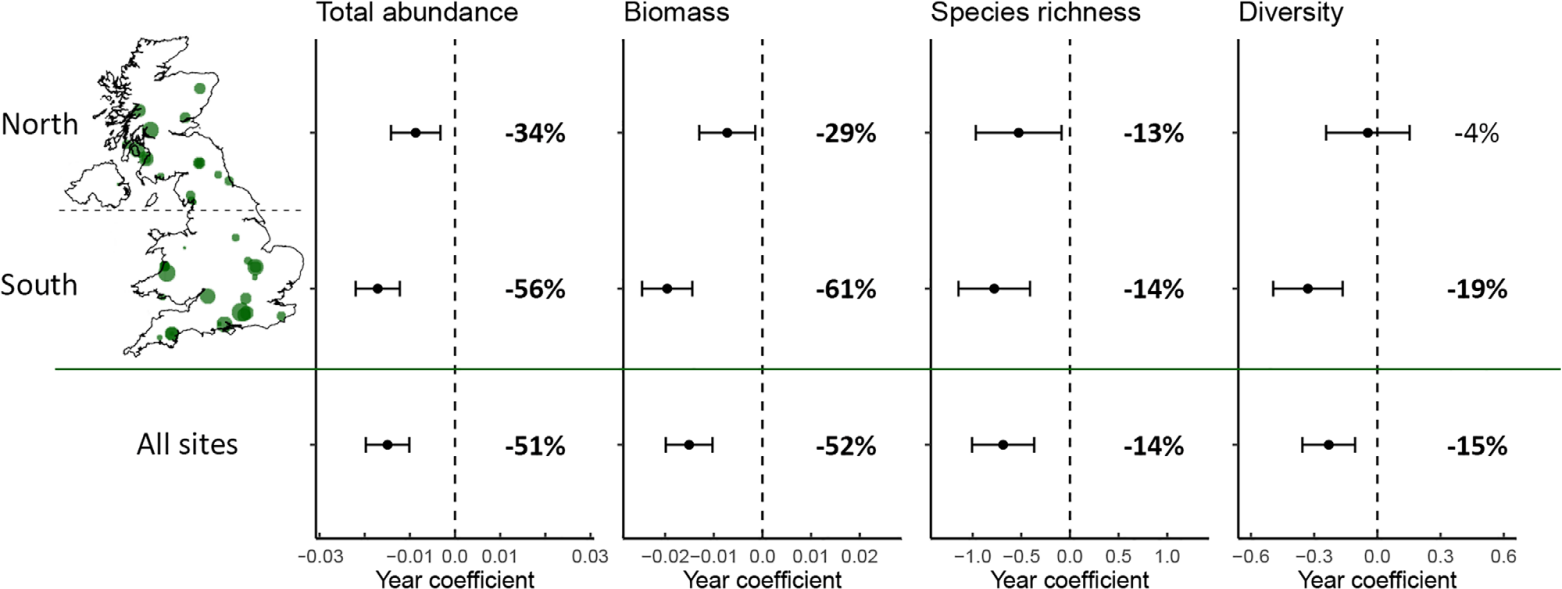
Year coefficients from the parametric components of GAMMs with 95% confidence intervals for four response variables in broadleaf woodland sites in the north, south and all sites combined. Percentage change over 1968–2016 is shown next to the error bars with significant trends in bold. Map displays the location of sites used in the models with size of points proportional to the time‐series length of the trap. Dashed lines show no change (i.e. zero coefficient). Note that coefficients are for different measures of moth community attributes

The decline in each of the four response variables in broadleaf woodlands was more severe in the south than in the north (Figure 3). All declines were significant apart from the change in diversity in northern broadleaf woodlands. In all cases, a non‐linear model outperformed a linear model (Table 1). In northern sites, abundance and biomass in broadleaf woodlands both increased in the first half of the time series before declining to a lower level than their starting points. In the south, abundance and biomass declined consistently (Figure S6a,b). Poptrend analysis showed that both the initial increase and subsequent decline were statistically significant (Figure S7). In contrast, abundance in southern broadleaf woodlands underwent a continuous significant decline throughout almost the entire time series.

### Effect of deer damage in broadleaf woodland sites

The probability of deer damage within woodland (as estimated using the online tool) sites ranged from 0.55 to 0.86. Of the 42 sites used in the analysis, eight could not be included as their location was outside the scope of the online tool. There was no interaction between year and deer damage likelihood for any of the four response variables: abundance (*χ* ^2^ = 3.4, *p* = 0.063), biomass (*F* = 3.7, *p* = 0.54), species richness (*F* = 0.091, *p* = 0.76) or diversity (*F* = 1.6, *p* = 0.21).

### Species‐specific analysis

A total of 10,963,959 moths belonging to 427 species were included in models across 384 sites, 49 years, and 4328 site‐years. The mean expected species‐specific trend when including all sites was a decline of 40% which differed significantly from zero (*t*‐test, df = 426, *t* = 58.6, *p* < 0.001). There was a significant effect of habitat (LRT, *χ* ^2^ = 122.4, *p* < 0.001), region (LRT, *χ* ^2^ = 32.5, *p* < 0.001) and region within broadleaf woodlands (LRT, *χ* ^2^ = 38.1, *p* < 0.001) on the mean species trend (Figure S8). These broadly matched the findings of the general analysis, with stronger declines in the south. The average species trend in arable and upland sites was stable whereas all other sites showed a significantly declining trend. When considering all UK sites, trends were significantly affected by feeding guild (*F*‐test, *F* = 4.99, *p* < 0.001, Figure S9a) but not for Ellenberg value for light (*F*‐test, *F* = 2.42, *p* = 0.12, Figure S9b). Species feeding on lichens increased significantly, those feeding on conifers remained stable and all six of the other groups declined significantly.

**FIGURE 4:**
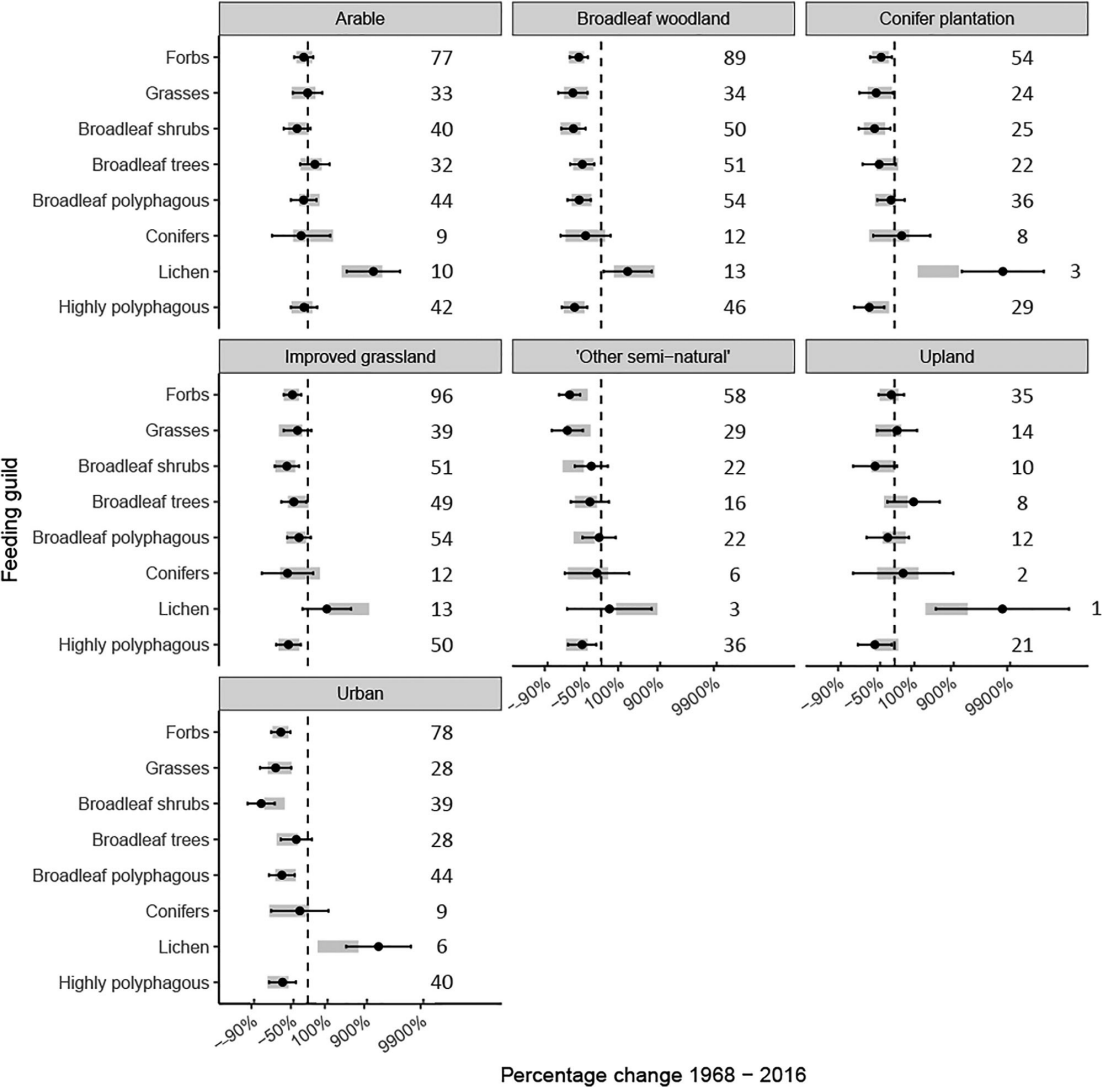
Percentage changes in moth abundance in species‐specific models 1968–2016 for species in eight feeding guilds in seven habitat types. Points and whiskers show estimated marginal means and 95% CIs from a LMM that specifies an interaction between feeding guild and habitat. Grey bars show the 95% CIs from a model that specifies no interaction effect between feeding guild and habitat. An LMM found that there was a significant interaction effect between habitat and feeding guild (*p* < 0.001). Dashed line shows zero percent change in each plot. The numbers to the right of each bar shows the number of moth species included in that group

There was a significant interaction between feeding guild and habitat (LRT, χ^2^ = 90.3, *p* < 0.001; Figure 4), region (LRT, *χ* ^2^ = 19.6, *p* < 0.006; Figure S10a) and region within broadleaf woodlands (LRT, *χ* ^2^ = 38.3, *p* < 0.001; Figure S10b). Post hoc analysis suggested that the interactions were driven by lichen feeders, grass feeders, broadleaf tree feeders and broadleaf shrub feeders (Figure 4). Lichen feeders had more positive trends than expected in urban areas and more negative trends in improved grassland. Broadleaf shrub feeders had more positive trends than expected in ‘other semi‐natural’ and more negative in urban. Broadleaf tree feeders had more positive trends than expected in uplands. Grass feeders had more negative trends than expected in the north and in northern woodlands (Figure 10b). Species feeding on broadleaf trees and shrubs had more positive trends than expected in northern woodlands. Contrary to our hypothesis, species that feed on forbs and shrubs were no more likely to decline in woodlands than those that feed on grasses, trees or lichens. No significant interactions were found for Ellenberg value for light (Table S3).

## DISCUSSION

Our results show that between 1968 and 2016 in the United Kingdom, there were significant declines in total moth abundance (−34%) and biomass (−39%), while species richness remained unchanged and diversity increased (+10%). The steeper decline in total biomass compared to total abundance is likely due to steeper declines in larger‐bodied moths, as was shown by Coulthard et al. (2019). The stability of species richness and the increase in diversity is supported by findings that show occupancy of moth species in the United Kingdom has increased since the 1960s (Dennis et al., 2019; Outhwaite et al., 2020; Randle, 2019). When taking region into account, species richness had a significantly positive trend of 9% in the north while richness in the south did not change significantly (Figure 1). This corroborates the finding that much of the increase in occupancy has accompanied a northwards spread (Fox et al., 2021). A similar pattern of change was also reported in Finland (Antão et al., 2020) and is likely due to species range expansions, made possible by climate change, counteracting a loss in abundance. The decline in abundance in broadleaf woodland (−51%) was more severe than that of any other habitat, including intensively farmed habitats: arable (−7%, non‐significant) and improved grassland (−28%). Broadleaf woodland also showed the greatest and most consistent declines in all four community attributes examined (abundance, biomass, species richness and diversity).

In all four response variables, trends were more negative in the south than the north. However, it should be noted that trends in total abundance in the north were very sensitive to the exclusion of single sites (see Supporting Information: Influential sites and Figure S11). The removal of a single influential outlier site (in the improved grassland category) from the model resulted in the abundance trend in the north dropping from −25% to −40% and therefore matching the south in magnitude of decline (Figure S11b). This means that the previously reported differing rates of decline in moth abundance between the north and south (Conrad et al., 2006; Fox et al., 2021) may not represent a genuine difference, but may rather be a result of the influence of a single long‐running site which underwent a very large increase in abundance, likely due to the development of a poplar tree plantation around the trap (see Figure S12 for more information about the large increase in abundance at this site). In support of more severe abundance declines in the south is the fact that the same pattern emerged in broadleaf woodland (Figure 3) and this was robust to the exclusion of single sites (Figure S11d). Unfortunately, the difference in trends between north and south could not be tested across all habitats due to a low number of sites in some habitats and a lack of northern sites for arable habitat and southern sites for upland habitats. In the south, the decline in biomass was more severe than the decline in abundance, while in the north, the opposite was true (Figure 1), indicating that larger‐bodied moths have fared worse in the south but better in the north.

### Decline in biomass

Declines in moth biomass are a direct result of declines in abundance and have the same implications. Moths are an important food source for other organisms in both their larval and adult form, and a loss of 39% of biomass is clearly detrimental to species that prey on them such as bats, insectivorous birds and predatory invertebrates. For example, a link between insect decline and insectivorous bird decline has been shown for European species (Bowler et al., 2019; Møller, 2019).

Our finding that biomass had declined by 39% partially agrees with Macgregor et al. (2019) who (after corrections to the manuscript were submitted in 2021) found that although there was no difference in mean biomass between the first and last decades of the time series, there was nonetheless a significant negative trend as revealed by linear regression. Using figures given in their supplementary information (biomass in year *t* + 1 = biomass in year *t* × e^−0.006^), it can be calculated that they found a decline of 6% per decade: the equivalent of a decline of 25% from 1968 to 2016. The discrepancy between the findings of Macgregor et al. (2019) and the figure presented here is due partly to the smaller selection of sites used by Macgregor. When we repeated our own analysis described above for the 34 long‐running sites used by Macgregor et al. (2019), we found a decline in biomass of 30% rather than 39% when including all sites with at least 3 years run time (see R code in Supporting Information S1). This discrepancy highlights the limitations of deriving national population trends from a limited number of sites. Each site has its own idiosyncrasies and management history, so cannot offer much insight into national trends on its own. However, the more sites included in the analysis, the better the model will represent the true national trend. This is why we chose to include as many sites as possible, even those with short time series length.

Differences in the modelling technique also explain some of the discrepancy in results. Macgregor et al. (2019) used as response variable, in the linear regression, the geometric mean biomass per trap which does not account for site turnover and the large variation in catch sizes between sites. To account for these effects, they performed two mixed effects models, with site‐level random intercepts, splitting the time series into two at 1976 where there was a peak in biomass. They found no significant change from 1967 to 1976 and a significant decline of 28% from 1976 to 2017. While Macgregor et al. (2019) stated that there has been no change in moth biomass between the first and last decades in their study, this statement derives from a *t*‐test that does not account for trap turnover. When using a more appropriate mixed effects model, they found a significant decline in moth biomass occurring mainly after 1976, which is consistent in magnitude to the one we observed for the same subset of sites (28% compared to 30% decline). We therefore disagree with Macgregor et al. (2019) where they state that moth biomass ‘lacks a clear trend’: when all available sites are included and modelled including site‐level random effects, we found a significant decline of 39%.

### Moth decline in broadleaf woodland

Our findings of severe moth declines in woodlands echo those of Roth et al. (2021) who found that from 1978 to 2018 moths in woodlands across Germany had declined by 53% in abundance, 57% in biomass and 38% in species richness, compared to our findings of declines of 51%, 52% and 14%, respectively, from 1968 to 2016. The decline we found in both species richness and diversity in woodlands is especially concerning given no other habitat experienced these declines, and the fact that UK broadleaf woodland extent has increased over the same time (Hopkins & Kirby, 2007). However, despite an increasing wooded area, the UK continues to lose mature and ancient woodland to development, with the habitat quality of newly planted areas not matching what has been lost (Reid et al., 2021). We predicted that the decline in broadleaf woodlands could be due to structural changes that have resulted from the cessation of active management and increased deer density: namely, shading and intensive browsing. We hypothesised that these changes would have reduced the quantity of forbs, shrubs and shade‐intolerant plants, that are more vulnerable to browsing and shading, leading to a decline in moth species feeding on them compared to moths feeding on trees, grasses, lichens and shade‐tolerant plants. However, no such trend was evident (Figure 4). Heavy browsing by deer typically results in a reduction of specific plant species such as bramble (*Rubus fruticosus* agg.), oak (*Quercus* sp.) and willow (*Salix* sp.) and the increase of grasses and unpalatable species such as bracken (*Pteridium aquilinum*) (Gill & Beardall, 2001; Kirby, 2001). Long‐term changes in plant species composition with UK woodlands are consistent with heavy browsing (Amar et al., 2010). Despite this, we found no sign that moth species specialising on these plants were any more or less likely to decline in broadleaf woodland than in the United Kingdom as a whole (Figure S13). It is possible that such deer activity also negatively affected species that feed on trees by removing the young seedings from the understory. In addition, some moth species prefer to feed on young, sun‐exposed trees or trees in open situations rather than mature specimens or trees in shaded understories (Waring & Townsend, 2017). For such species, an increase in shadiness would also negatively affect species considered robust to shading in this study, but this level of detail was not included in our analysis as it would require very fine‐grained knowledge of species traits which are either not known or are not documented in any accessible form.

Whilst woodland butterfly and bird declines show evidence to support the shading and browsing hypothesis (Fartmann et al., 2013; Fuller et al., 2005; Sparks et al., 1996), nocturnal moths appear to be less negatively affected by shading. Compared to butterflies, adult moths are not dependant on sunlight for warmth but rather use muscular activity to raise their body temperatures (Clench, 1966). Merckx, Feber, et al. (2012) found that moth abundance and species richness was higher in more mature woodland compared to coppiced woodland, so we might expect the cessation of coppicing to have caused an increase rather than a decrease in abundance and diversity. In contrast, Roth et al. (2021) found that at a single coppice woodland site, species richness had increased over the same period in which richness had declined across forests across Germany. Whilst there is some evidence that moth abundance and species richness is reduced in woodlands exposed to grazing (Fuentes‐Montemayor et al., 2012), there is no record of historic management practises of woodlands across the RIS light‐trap network, so we could not test whether declines were predicted by canopy closure and deer density at the site level. We estimated the likelihood of deer damage for each woodland site based on site characteristics using an online tool described above but found no significant effect of estimated deer damage on rate of decline. While this tool provided the best estimates of deer damage currently available, it is only a static snapshot of deer browsing intensity and does not take into account how deer densities have grown over time, so it may not accurately capture the true changes in deer browsing intensity that have occurred at each of the RIS woodland moth‐trap sites.

The planting of exotic conifers in native broadleaf woodland sites that occurred post‐1968 (Rackham, 2003) could also have affected moth populations. However, we used land‐use data from 2015 and sorted any conifer‐dominant woodlands into a separate category, meaning that any site extensively converted to conifer‐dominated woodland before 2015 would have been excluded from the analysis of broadleaf woodlands. Furthermore, moth species that feed on conifers showed no increase in either broadleaf woodlands or conifer plantations, suggesting that the proportion of coniferous to broadleaf trees at the sites did not undergo any widespread changes during the study period.

There was some difference in the feeding guilds of moths most affected in the northern and southern woodlands. In the north, species feeding on broadleaf trees and shrubs tended to have more positive trends than expected if there were no interaction effect, and those feeding on grasses and forbs tended to have more negative trends than expected (Figure S10b). This suggests that woodlands in the north have become less open in structure. However, moths specialising on light‐loving plants were no more likely to decline in northern woodlands than those feeding on shade‐tolerant plants, meaning that support for the shading hypothesis is weak.

Finally, climate change is known to have contributed to the national decline in moths (Martay et al., 2017) and it is likely that this has driven at least part of the decline observed in woodlands. However, this cannot explain why the declines have been worse in broadleaf woodland compared to other habitats. Indeed, we might expect the shade provided by woodlands to help buffer against the effects of climate change (De Frenne et al., 2013). However, the interaction between climate, habitat and moth abundance trends remains to be tested.

### Moth populations in farmland

Despite the widespread intensification of UK farmland since the late 1960s, it is surprising that species diversity has increased in these habitats and there has been no decline in species richness. This may be due, in part, to the warming climate allowing species to spread into new areas, with the new arrivals balancing out losses due to habitat degradation; a conclusion drawn by Fox et al. (2014) to explain similar observations. It may also be the case that much of the damage to moth populations through agricultural intensification had already taken place before the start of this time series. A single Rothamsted trap that ran during the 1930s and 40s reported a 71% decline in abundance when the trap was restarted in the 1960s (Woiwod & Gould, 2008). This decline was attributed to the rapid land‐use changes that occurred during the 1950s, including widespread habitat loss due to agricultural mechanisation and the introduction of synthetic herbicides and insecticides. While intensification of agriculture did continue well beyond 1968 (Robinson & Sutherland, 2002), the introduction of agri‐environment schemes (AESs) and their subsequent expansion in the 2000s (Grice et al., 2006) may have mitigated declines in farmland to some extent. Experimental studies have shown that AES features such as sown field margins and sympathetically managed hedgerows are effective at enhancing local moth abundance and diversity (Alison et al., 2016; Merckx, Marini, et al., 2012; Staley et al., 2016). However, if this were the case, we would expect to see a decline in response variables up until the early 2000s with a subsequent recovery, but the long‐term trends show no evidence of this pattern in either arable land or improved grassland (Figure S4). While species richness appears stable in farmland, abundance declined significantly in improved grassland and biomass declined significantly in both arable land and improved grassland. Again, the non‐linear trends show no sign of recovery in recent decades. These findings match similarly muted responses of bird populations to widespread AES adoption in recent years (Chamberlain, 2018), suggesting that such schemes either need to be improved or adopted on a much larger scale, or both, to be effective (Walker et al., 2018).

### Moth decline in other habitats

Declines in abundance and biomass were also detected across all other habitat types apart from ‘other semi‐natural’ in which there was no significant decline in abundance (although there was a significant decline in biomass of 43%). After broadleaf woodlands, the most severe declines occurred in urban areas, with a decline of abundance and biomass −44% and −46%, respectively. As our habitat categorisation process only used land‐use data from 2015, this means that sites classed as ‘urban’ also included those that had urbanised at some point between 1968 and 2015. It is therefore likely that habitat loss and light pollution resulting from the urbanisation that occurred over this period has contributed to these large declines. Despite these losses, species diversity in urban sites increased by 24% over the same period. Species feeding on lichen did especially well in urban areas (Figure 4), likely due to the reduction in air pollution over this time which promoted the growth of lichens (Gilbert, 1992): although note that this result is based on only six species so does not have the same robustness as other feeding guilds.

The final three habitat types, conifer plantation, ‘other semi‐natural’ and upland sites all had low numbers of sites used in the analysis (12, 19 and 15 sites each, respectively), so results should be interpreted cautiously. The trend estimate for ‘other‐semi‐natural’ habitat was very sensitive to the removal of single sites, but upland and conifer plantations were robust (Figure S11c). Species feeding on broadleaf shrubs and those polyphagous on broadleaf shrubs and trees did especially well in ‘other semi‐natural’ habitats, while those feeding on grasses and forbs were more likely to have declined. This suggests that the development of scrub at these typically open sites may have contributed to the decline in moth biomass at these sites.

## CONCLUSIONS

Here, we have shown that declines in moth abundance have occurred across most habitats and that declines in biomass have occurred across all habitat types in the United Kingdom from 1968 to 2016. We also show, for the first time, that despite national declines in abundance and biomass, species richness has remained stable and diversity has increased at the national level, with most of this increase in diversity occurring in urban and intensively farmed areas. We have shown that the most severe declines have occurred not in areas of intensive agriculture, but in broadleaf woodlands – especially in southern broadleaf woodlands. We found no evidence that species more likely to suffer from shading and browsing by deer had declined more in broadleaf woodland than other species, so the reasons for the more severe decline in this habitat compared to others remain unclear. Further research should investigate other potential drivers of moth decline in broadleaf woodlands, including invasive plant species and climate change.

## CONFLICT OF INTEREST

There are no conflicts of interest to declare among the authors of the manuscript.

## Supporting information


**Appendix S1.** Supplementary Information

## Data Availability

Data and R code to reproduce the analysis are available on the Rothamsted Research Repository under a Creative Commons Attribution 4.0 Licence (https://doi.org/10.23637/rothamsted.98852).
