# Supplementary material for: Moth declines are most severe in broadleaf woodlands despite a net gain in habitat availability
Source: Insect Conserv Divers. 2022 Apr 28;15(5):496–509. doi: 10.1111/icad.12578 (PMC9545439; doi:10.1111/icad.12578)
Supplement: Supplementary file 1 — Appendix S1. Supplementary Information [file ICAD-15-496-s001.docx]

Supplementary information

# Methods


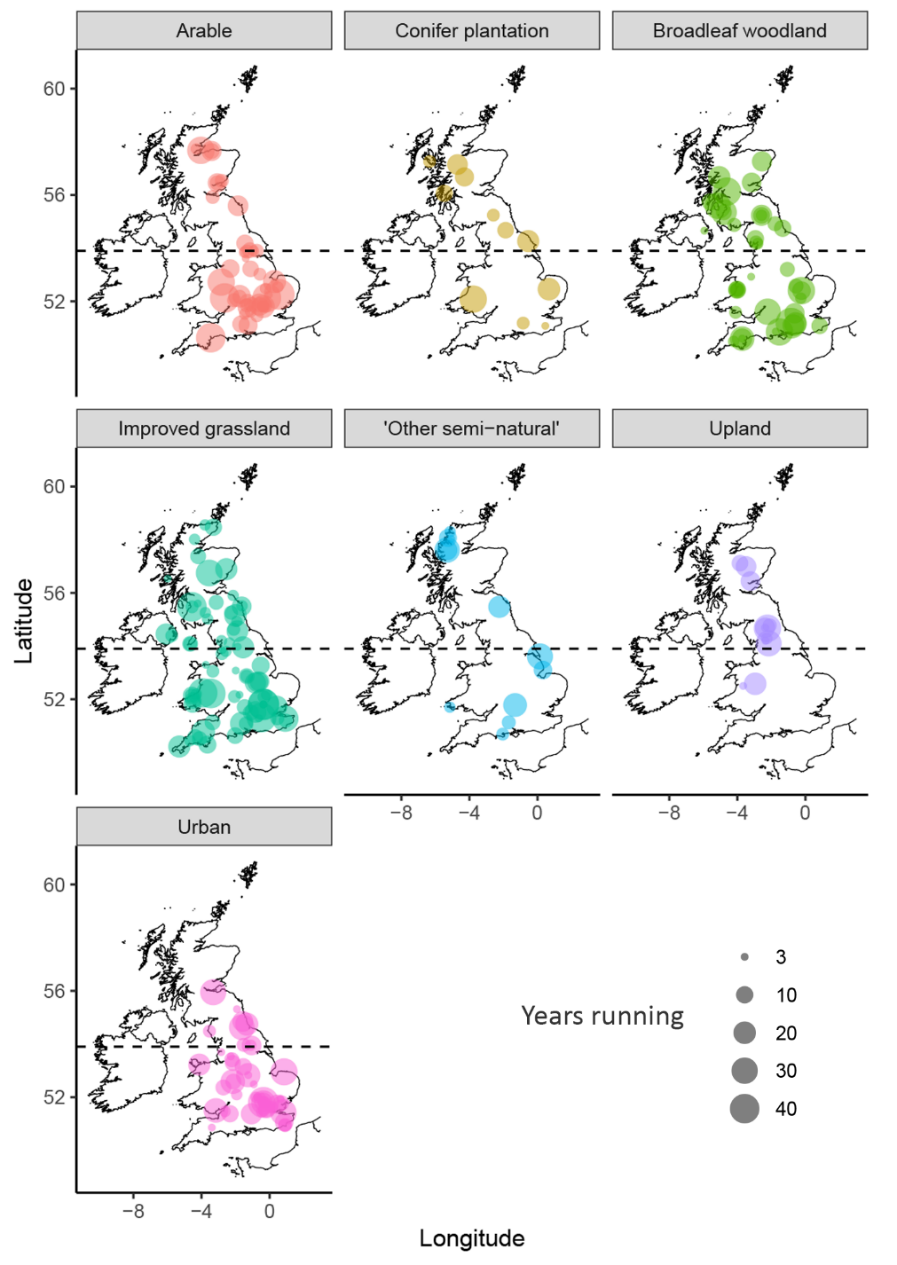


Fig S1. Location of the 266 RIS moth-trap sites within the UK used in the ‘general analysis’ section of this study across seven habitat types. The size of the point is proportional to the number of years the trap ran for between 1968 and 2016. The dashed line shows the 4500 Northings gridline that was used to divide the country into north and south.

Table S1. Habitat types used for analysis based on the dominant land cover type within 500m radius of the trap, according to the LCM 2015. The table shows number of sites in each LCM 2015 land cover type. The numbers not in brackets show the number of sites used in the ‘general analysis’ (running for at least three ‘complete’ years with no gaps in recording of more than two weeks from 1 April to 31 October or more than four weeks from 1 November to 31 March). The number in brackets shows the number of sites used in the ‘species-specific’ analysis. These sites ran for at least three years, including incomplete years. The number of sites in each region (north or south) is shown, using 4500 N gridline on the British National Grid as a dividing line.

| Altitude class | LCM 2015 | North | South | LCM total | Habitat type | Habitat total |
| --- | --- | --- | --- | --- | --- | --- |
| Upland (300 m or higher) | Acid grassland | 5 (5) | 0 (1) | 5 (6) | Upland | 12 (15) |
|  | Bog | 2 (2) | 0 | 2 (2) |  |  |
|  | Coniferous woodland | 1 (1) | 1 (1) | 2 (2) |  |  |
|  | Improved grassland | 2 (3) | 1 (2) | 3 (5) |  |  |
| Lowland (lower than 300 m) | Arable and horticulture | 10 (11) | 50 (65) | 60 (76) | Arable | 60 (76) |
|  | Coniferous woodland | 7 (8) | 4 (4) | 11 (12) | Conifer plantation | 11 (12) |
|  | Broadleaved woodland | 18 (22) | 24 (29) | 42 (51) | Broadleaf woodland | 42 (51) |
|  | Improved grassland | 29 (44) | 47 (85) | 76 (129) | Improved grassland | 76 (129) |
|  | Acid grassland | 1 (1) | 0 (1) | 1 (2) | ‘Other semi-natural’ | 12 (19) |
|  | Calcareous grassland | 0 | 1 (1) | 1 (1) |  |  |
|  | Heather | 1 (2) | 1 (2) | 2 (4) |  |  |
|  | Heather grassland | 0 | 4 (5) | 4 (5) |  |  |
|  | Littoral sediment | 0 | 1 (1) | 1 (1) |  |  |
|  | Neutral grassland | 0 | 1 (1) | 1 (1) |  |  |
|  | Saltmarsh | 0 | 1 (3) | 1 (3) |  |  |
|  | Supra-littoral rock  Supra-littoral sediment | 0  0 | 1 (1)  0 (1) | 1 (1)  0 (1) |  |  |
|  | Urban | 1 (1) | 5 (9) | 6 (10) | Urban | 53 (82) |
|  | Suburban | 8 (14) | 39 (58) | 47 (72) |  |  |
|  | **Total** | 89 (119) | 177 (265) | 266 (384) |  | 266 (384) |

Table S2. Moth species traits used in this study with descriptions of the levels within each trait.

| Trait | Level of trait | Notes |
| --- | --- | --- |
| Feeding guild | Conifers | Feeds exclusively on coniferous trees and shrubs |
|  | Broadleaf shrubs | Feeds exclusively on broadleaf shrubs. In this case, a ‘shrub’ is a woody plant that does not exceed 15 m in maximum height and is typically not a canopy-forming plant in mature woodland. E.g., hawthorn, blackthorn, hazel, elder, privet, rowan, rose, bramble, currant, spindle, heather, gorse, broom, grey willow. |
|  | Broadleaf trees | Feeds exclusively on broadleaf trees. In this case, a ‘tree’ is a woody plant that exceeds 15 m in maximum growth and is often canopy-forming when growing in mature woodland. E.g., ash, beech, oak, lime, elm, hornbeam, birch, poplar, field maple, alder, white willow. |
|  | Broadleaf polyphagous | Polyphagous on both broadleaf trees and shrubs. |
|  | Forbs | Feeds exclusively on herbaceous plants, excluding grasses. |
|  | Grasses | Feeds exclusively on grasses, including reeds and sedges. |
|  | Highly polyphagous | Feeds on a range of both woody and herbaceous plants. |
|  | Lichens | Feeds exclusively on lichens or lichens and moss/algae. |
| Host plant Ellenberg value for light | 1 - 9 | Only for species that feed on three or fewer host plants: where there are more than one, the mean average value is used. |

**Estimating site-year completeness**

*The following can be replicated using Script_3_Turning_raw_data_to_site_indices supplied in the Supporting Information.*

Generalized Additive Models (GAM) were used following methods adapted from Dennis *et al.* (2013) to produce a 'site index' for each species-site-year combination (‘species-site-year’ from here). These site indices are an estimate of the annual sum of individuals caught had the trap been running for the entire flight-period. In this process, a 'completeness' score is estimated for each species-site-year with a value between 0 and 1. The site index is equal to the annual sum divided by completeness. Where there was continuous trapping through a species' flight-period (i.e., completeness = 1), the site index is equal to the annual sum.

In order to maximise the amount of data available, incomplete site-years were used, and missing counts were estimated using species flight periods. The data processing was as follows:

Step 1. ‘Derived’ nightly counts were calculated by dividing the total number of moths caught by the number of nights sampled. E.g., if one moth was caught over a four-night period, each night would be recorded as having caught 0.25 moths.

Step 2. Each calendar year was divided into 52 weeks. Within each week, a maximum ‘derived’ nightly count was extracted for each species at each site. This was rounded up to the nearest whole number.

Step 3. Flight periods for each species in each year in each region (north/south) were modelled using Generalised Additive Models (GAMs) with a Poisson error distribution. The maximum ‘derived’ nightly count was modelled as a function of calendar week. Flight periods were scaled so that the area under each curve summed to 1. Each calendar week for each species-year-region combination now has a value equivalent to the proportion of the flight period that occurred in that week.

Step 4. For each species-site-year, the calendar weeks in which the trap was running were extracted. As the dataset only included positive counts, true zero counts had to be inferred. For each night (or multi-night period) with a positive count for *any* species, all other species that were not caught that night were recorded as zero. Weeks for which no records existed for any species were classified as inoperative. For a week to be classed as operative, at least one count, of any species, had to occur.

Step 5. For each species-site-year, each calendar week was matched to the appropriate flight season for that species in that year in that region (north/south). The total proportion of the flight period sampled for that species-site-year was then estimated as follows. Each week within each species-site-year has already been allocated as either operative or inoperative (Step 4). Each operative week for each species-site-year was compared against the regional flight period for that species-year-region combination. For all operative weeks, the proportion of the flight period that occurred within each week was extracted (Step 3) and summed. The resulting figure is referred to from here on as ‘site-year completeness’ and takes a value from 0 to 1. Where a site was operative throughout the entire flight period for a species-site-year, the site-year completeness was equal to 1.

Step 6. ‘Site indices’ were calculated for each species-site-year by dividing the raw sum (total number of individuals of that species caught in that site-year) by the site-year completeness for that species. For example, if 100 individuals were caught, and site-year completeness was 0.8, then the resulting ‘site index’ would be equal to 125 This is the estimated number of individuals that would have been caught had the trap been continually running. Where site-year completeness is 1 (i.e., complete), then the site index is equal to the raw sum. Site indices were used as the response variable in the species-specific abundance models.

Note that a site-year can have different site-year completeness scores for different species within the same year. For example, if a trap is running throughout summer but is inoperative in autumn, species with summer flight periods will have site-year completeness score of 1, whereas species with flight periods overlapping autumn will have a site-year completeness score of < 1. In all models, to prevent spurious estimations, any species-site-year combination which had a completeness of less than 0.5 was omitted.

**Model quality-control**

In addition to only including site-years with a completeness of at least 0.5 and only using sites with at least three years of data, the following quality-control measures were implemented (these cut-off points for data quality were arrived at through trial-and-error). For each species-habitat combination, trends were only modelled if (1) the species was recorded in at least six sites, (2) the number of site-years in the model was greater than 100, (3) the number of individuals recorded in the time series was at least 200 and (4) the time series included both the first (1968) and final (2016) year. Despite these checks, the poptrend model occasionally failed to converge on some species-habitat or species-region combinations due to insufficient degrees of freedom. These models were also excluded.

# Results

## General trends


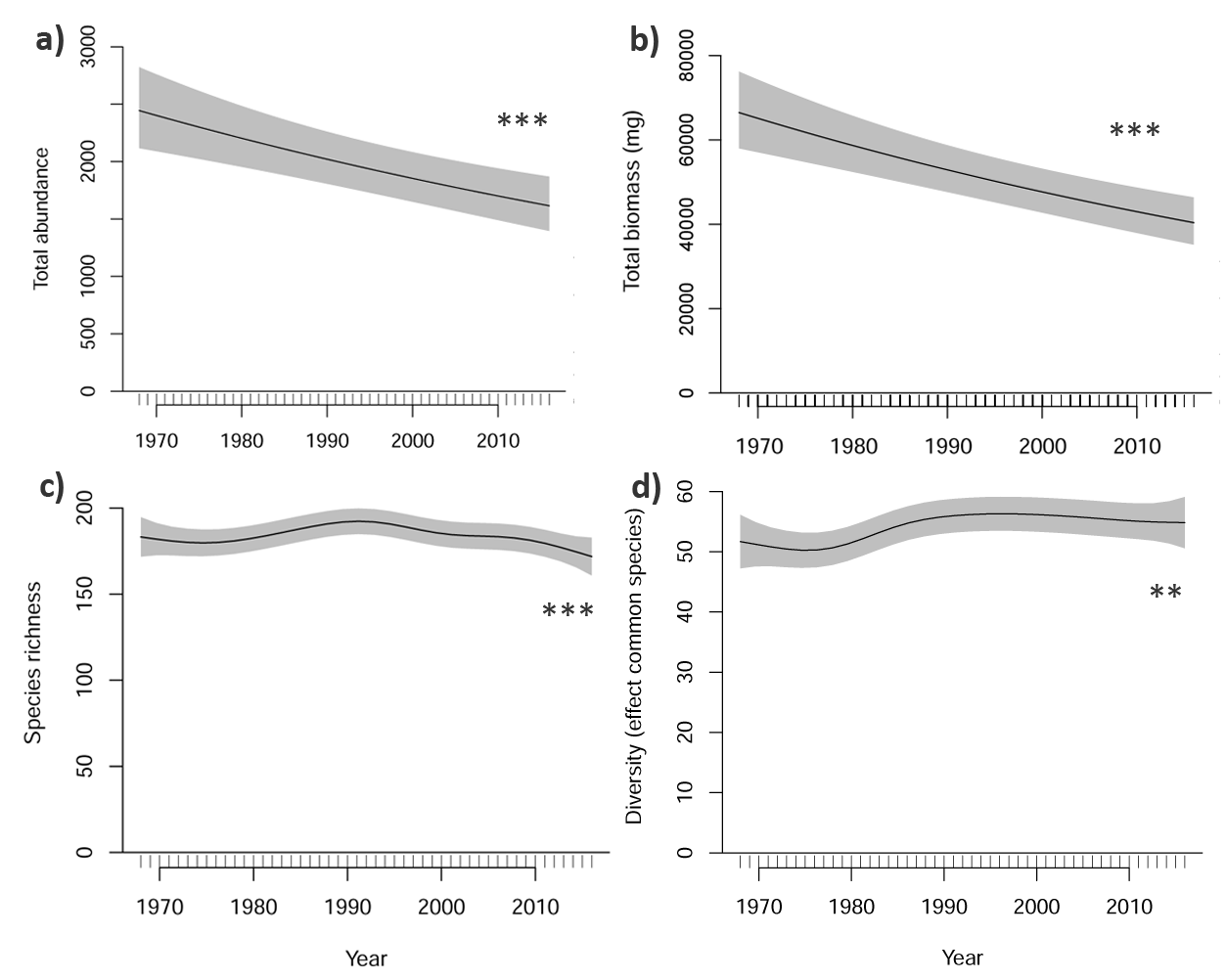


Fig S2. GAMM smooth predictions for (a) Total annual abundance, (b) biomass, (c) species richness and (d) Shannon diversity (effective common species) (d) from 1968 – 2016 across Great Britain. Solid black lines show GAMM predictions with grey ribbon showing 95% CIs, excluding random effects. Asterisks denote the significance of the smooth terms (p >= 0.05 ‘ns’, < 0.05 ‘*’, < 0.01 ‘**’, < 0.001 ‘***’).


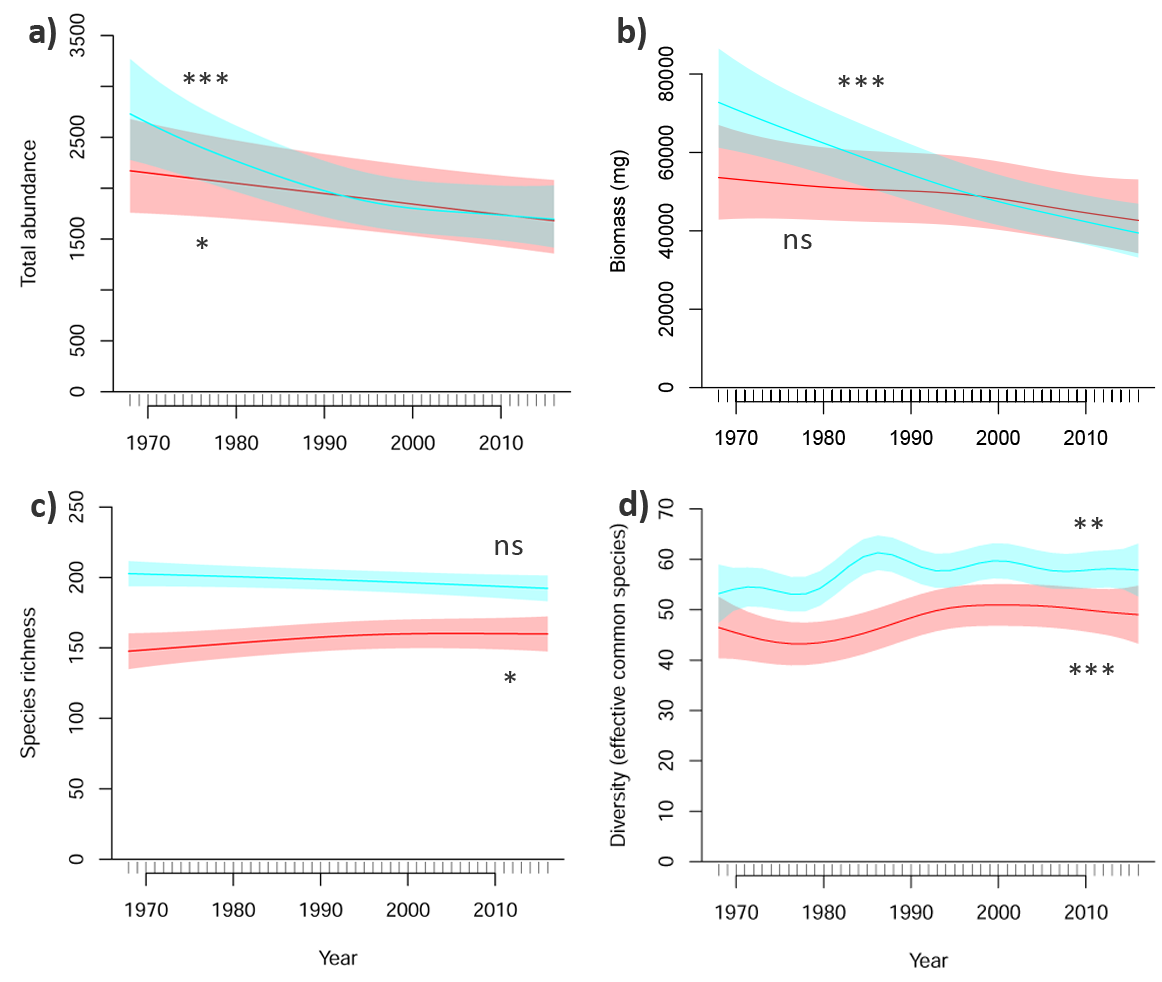


Fig S3. GAMM smooth predictions for (a) Total annual abundance, (b) biomass, (c) species richness and (d) Shannon diversity (effective common species) from 1968 – 2016 by northern (pink) and southern (turquoise) sites in Great Britain. Solid lines show GAMM predictions with coloured ribbons showing 95% CIs, excluding random effects. Asterisks denote the significance of the smooth terms (p >= 0.05 ‘ns’, < 0.05 ‘*’, < 0.01 ‘**’, < 0.001 ‘***’).


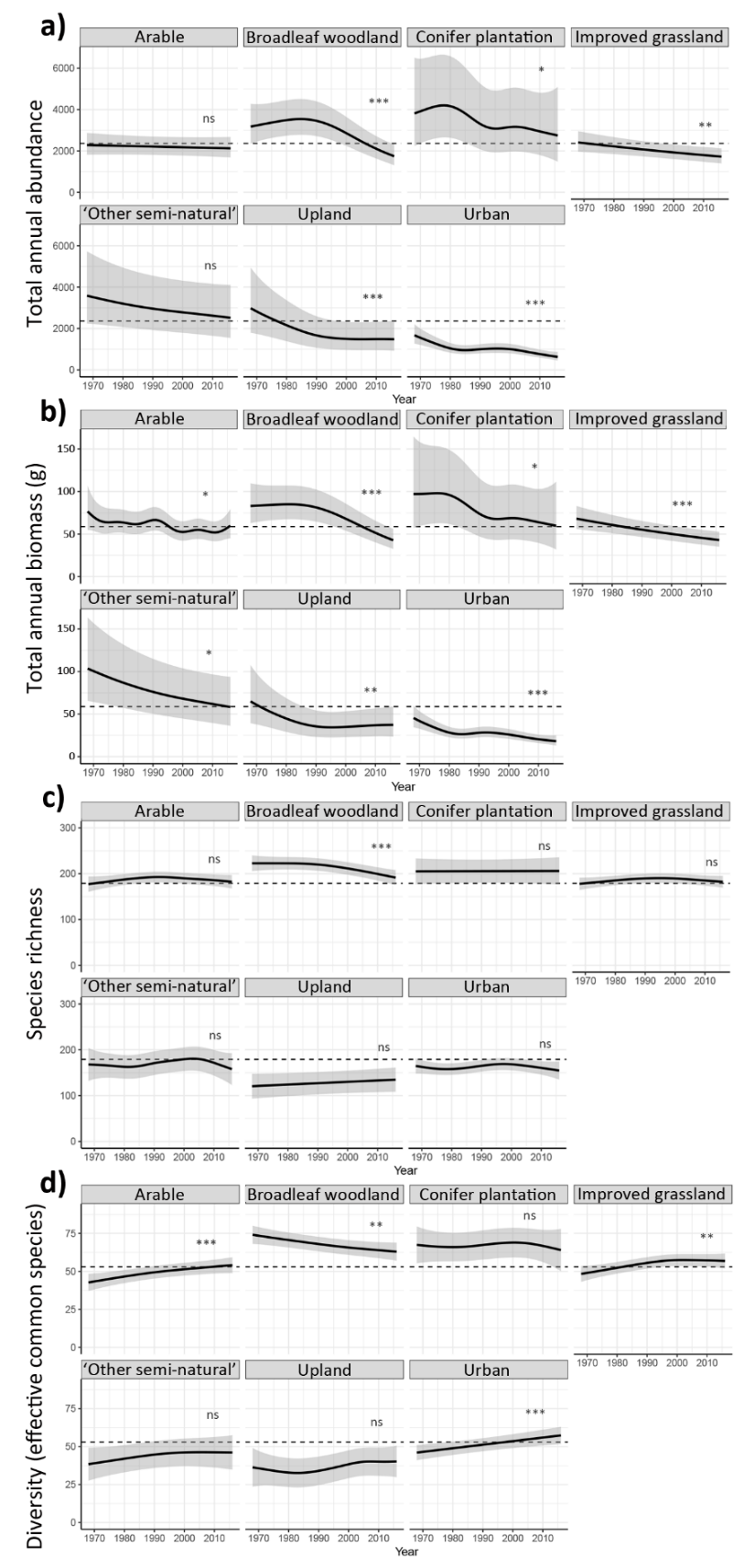


Fig S4. GAMM smooth predictions for (a) total annual abundance, (b) total annual biomass, (c) estimated species richness and (d) estimated Shannon diversity (effective common species) per site across seven habitat types 1968 – 2016. Solid black lines show GAMM predictions with shaded area showing 95% CIs, excluding random effects. Dashed lines show geometric mean prediction. Asterisks denote the significance of the smooth terms (p >= 0.05 ‘ns’, < 0.05 ‘*’, < 0.01 ‘**’, < 0.001 ‘***’).


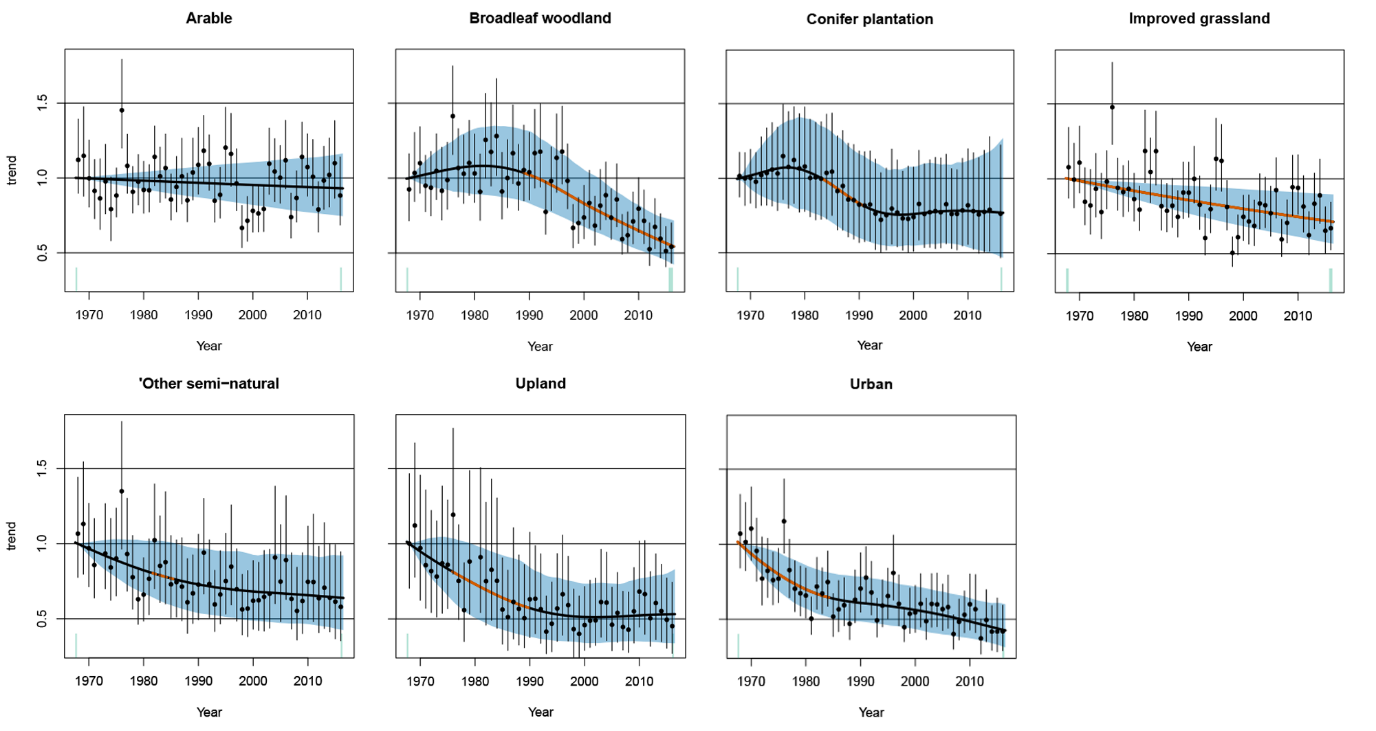


Fig S5. Population index for moths across seven habitat types 1968 - 2016 showing yearly mean and 95% CIs (solid line and blue shaded area respectively). Annual random effects with 95% CIs are shown by points and whiskers. Significantly decreasing short term trends at the 5% level are coloured orange, respectively. Models were run and plotted using the poptrend R package.


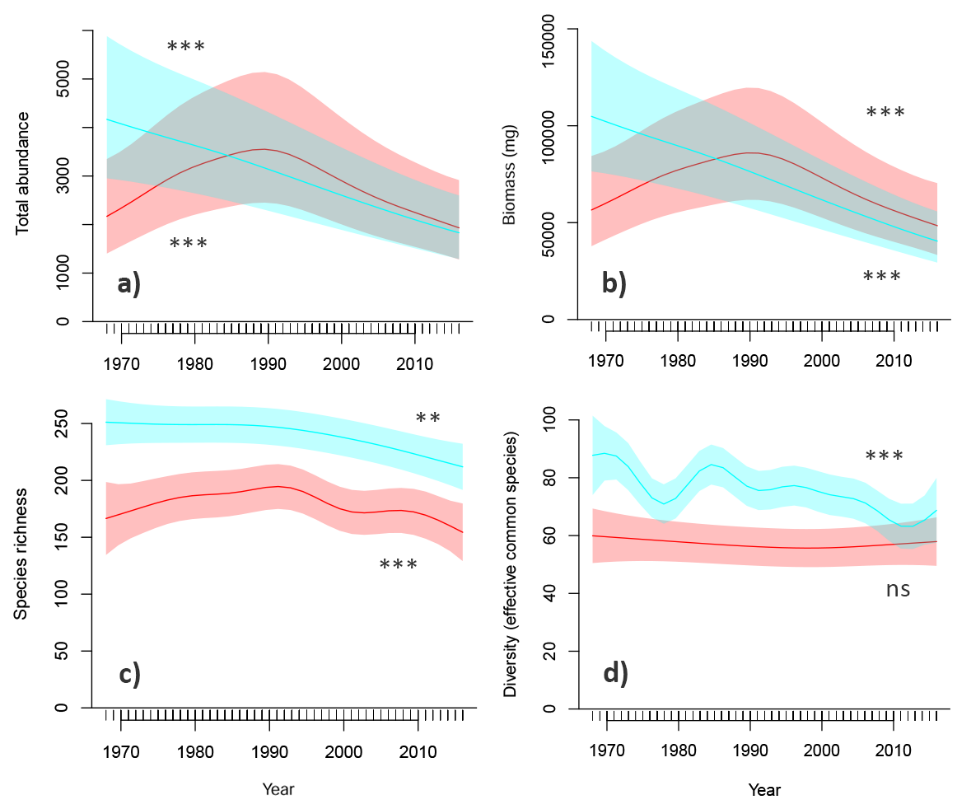


Fig S6. GAMM smooth predictions for broadleaf woodland sites only. (a) Total annual abundance, (b) biomass, (c) species richness and (d) Shannon diversity (effective common species) from 1968 – 2016. Solid lines show GAMM predictions with coloured ribbons showing 95% CIs, excluding random effects. Blue = south, pink = north. Asterisks denote the significance of the smooth terms (p >= 0.05 ‘ns’, < 0.05 ‘*’, < 0.01 ‘**’, < 0.001 ‘***’).


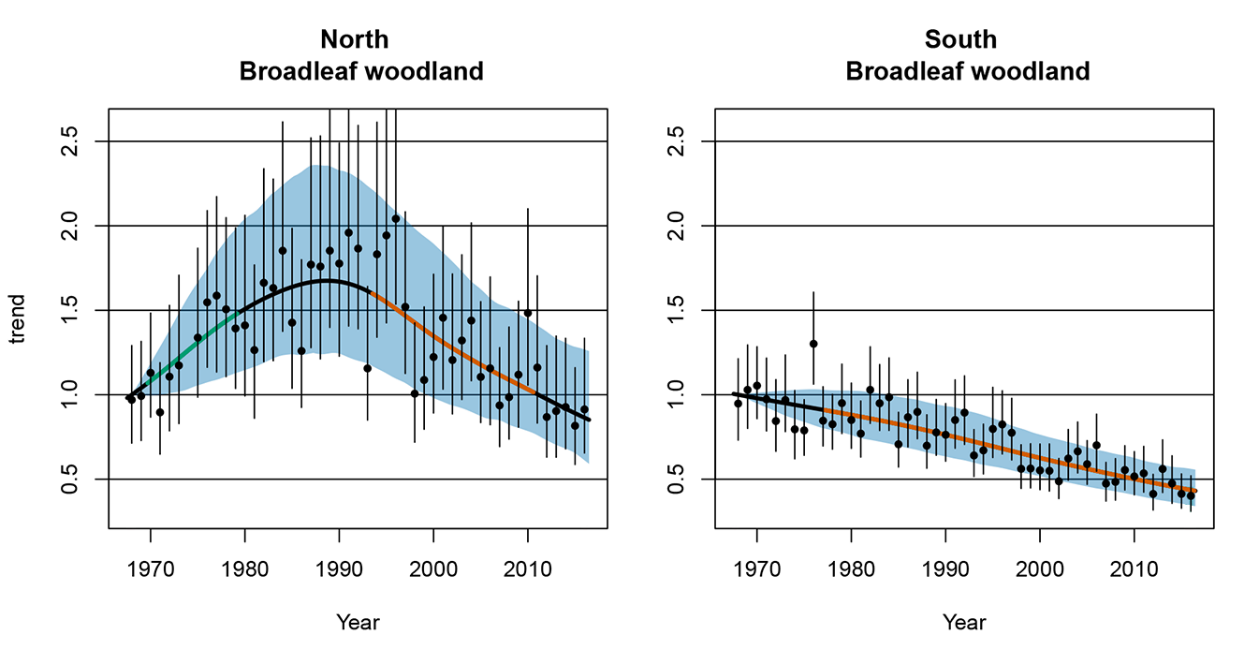


Fig S7. Population index for moths in broadleaf woodlands in the north and south of the UK 1968 - 2016 showing yearly mean and 95% CIs (solid line and blue shaded area respectively). Annual random effects with 95% CIs are shown by points and whiskers. Significantly increasing or decreasing short term trends at the 5% level are coloured green and orange, respectively. Models were run and plotted using the poptrend R package.


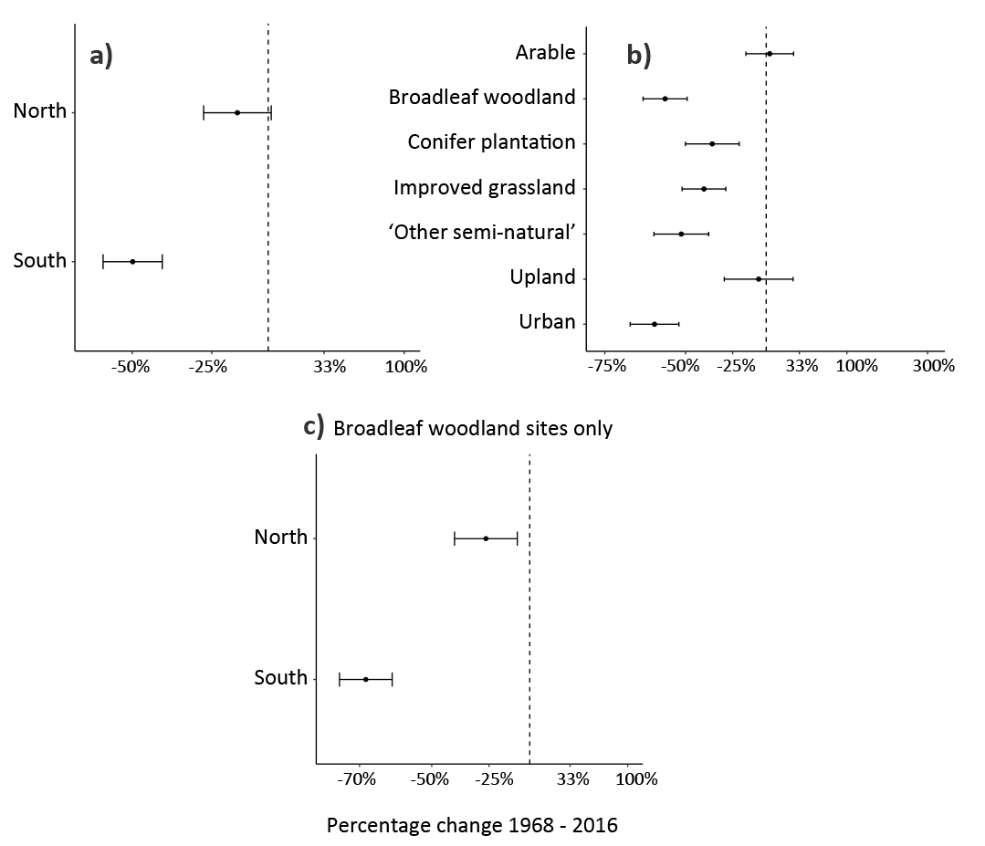


Fig S8. Percentage changes in abundance 1968 – 2016 using species-specific models according to (a) region, (b) habitat and (c) region for broadleaf woodland sites only. Points show estimated marginal means and 95% CIs are shown by error bars. Dashed lines show zero percent change in each plot.


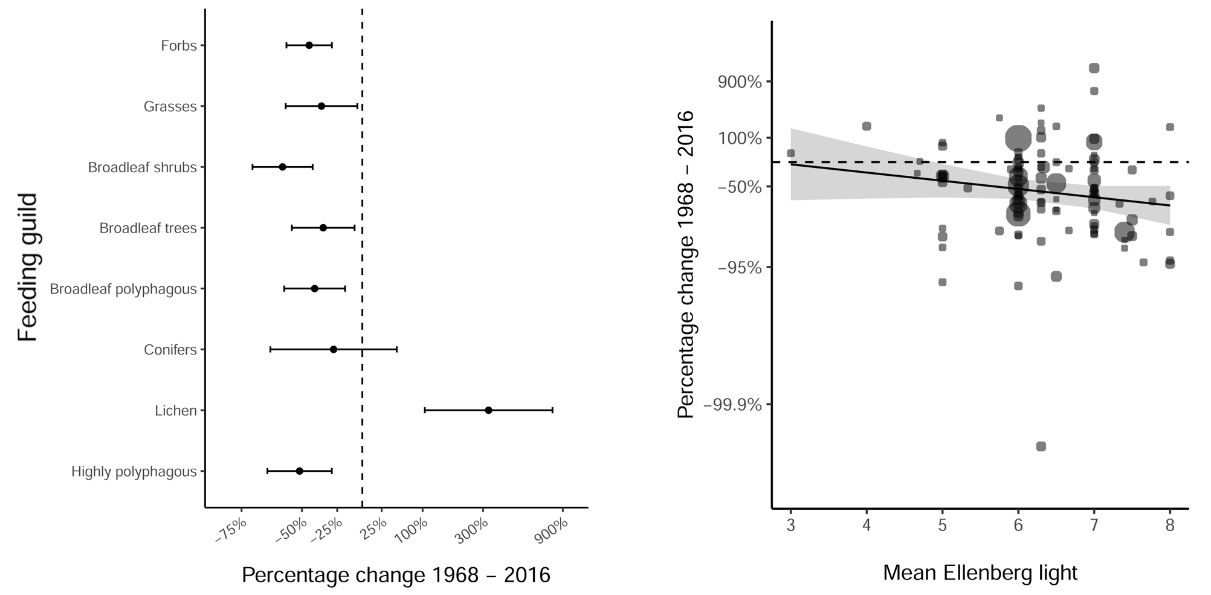

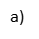

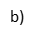


Fig S9. Percentage change in abundance 1968 – 2016 according to species (a) feeding guild and (b) Ellenberg value for light of the moth’s hostplant. (a) Points show estimated marginal means and 95% CIs are shown by error bars. (b) Higher Ellenberg value means a more light-loving hostplant. Estimated marginal mean is shown by the solid regression line and 95% CI is shown by grey ribbon. Each point represents one species with the size scaled to the log-transformed sample size for that species. There was a significant effect of feeding guild (p < 0.001) but not of Ellenberg light value on trend (p = 0.12). Dashed lines show zero percent change in both plots.

## Species-specific analysis


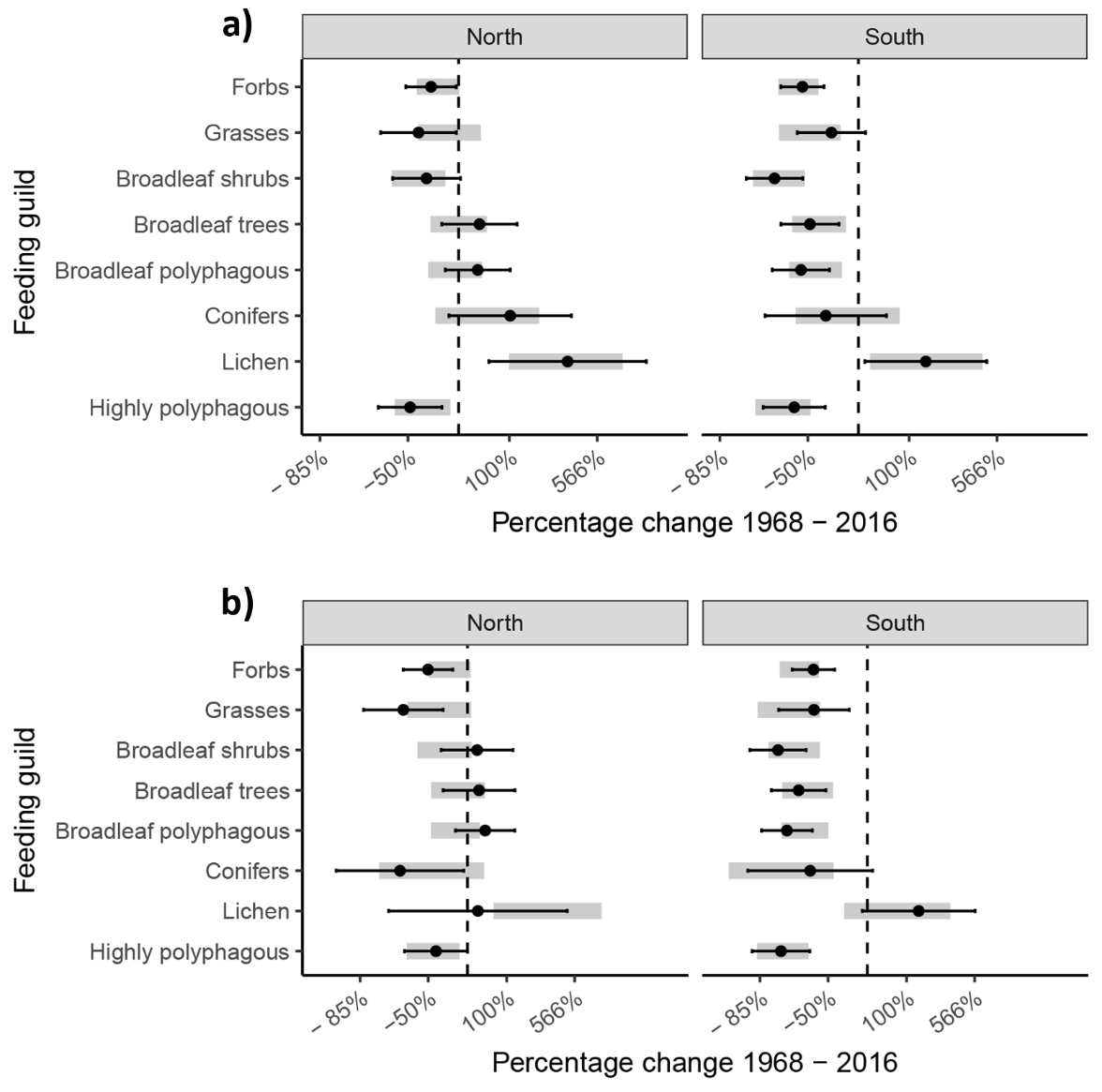


Fig S10. Percentage changes in abundance 1968 – 2016 using species-specific models for species in eight feeding guilds in both regions for (a) all sites and (b) for broadleaf woodland sites only. Points show estimated marginal means and error bars show 95% CIs from a LMM that specifies an interaction between feeding guild and region. Grey bars show the 95% CIs from a model that specifies no interaction effect between feeding guild and region. An LMM found that there was a significant interaction effect (p = 0.010) Dashed line shows zero percent change in each plot.

Table S3. Significance of main terms from a linear mixed effect model that estimated moth species trends as a function of various explanatory variables related to habitat and location. ΔAIC are shown comparing the full model to a reduced model with the main explanatory term removed. Where there is an interaction between two terms, this is compared to a model in which the effects are both present but not interacting. Asterisks denote the significance of the terms (p >= 0.05 ‘ns’, < 0.05 ‘*’, < 0.01 ‘**’, < 0.001 ‘***’).

| Explanatory variable(s) | ΔAIC | χ^2^ or *F* | Test statistic | P |
| --- | --- | --- | --- | --- |
| Habitat | 110.4 | 122.37 | χ ^2^ | <0.0001 *** |
| Region | 30.5 | 32.50 | χ^2^ | <0.0001 *** |
| Feeding guild | 20.2 | 4.99 | *F* | <0.0001 *** |
| Feeding guild*habitat | 6.3 | 90.31 | χ^2^ | <0.0001 *** |
| Feeding_guild*region | 5.6 | 19.61 | χ^2^ | 0.0065** |
| Ellenberg_light | 0.44 | 2.42 | *F* | 0.12 |
| Ellenberg_light*habitat | -4.9 | 7.18 | χ^2^ | 0.30 |
| Ellenberg_light*region | -0.96 | 1.04 | χ^2^ | 0.31 |
|  |  |  |  |  |
| Broadleaf woodland sites only | | | | |
| Region | 36.1 | 38.09 | χ^2^ | <0.0001 *** |
| Feeding_guild*region | 24.2 | 38.27 | χ^2^ | <0.0001 *** |
| Ellenberg_light*region | -1.95 | 0.05 | χ^2^ | 0.83 |

**Influential sites**

When the site named Auchincruive II was removed from the analysis, the total abundance trend in the north went from -25% to -40%. Trends in the south did not show such sensitivity (Fig. S11b). Auchnincruive II, a Scottish site operating for 40 years, underwent a 730% increase in total abundance, which appears due to a poplar plantation being planted around this site in the early 1990s (Fig. S12). Given the atypical nature of Auchincruive II, it is possible that this and previous analyses (Conrad *et al.*, 2006; Fox *et al.*, 2013) have underestimated the decline in the north.


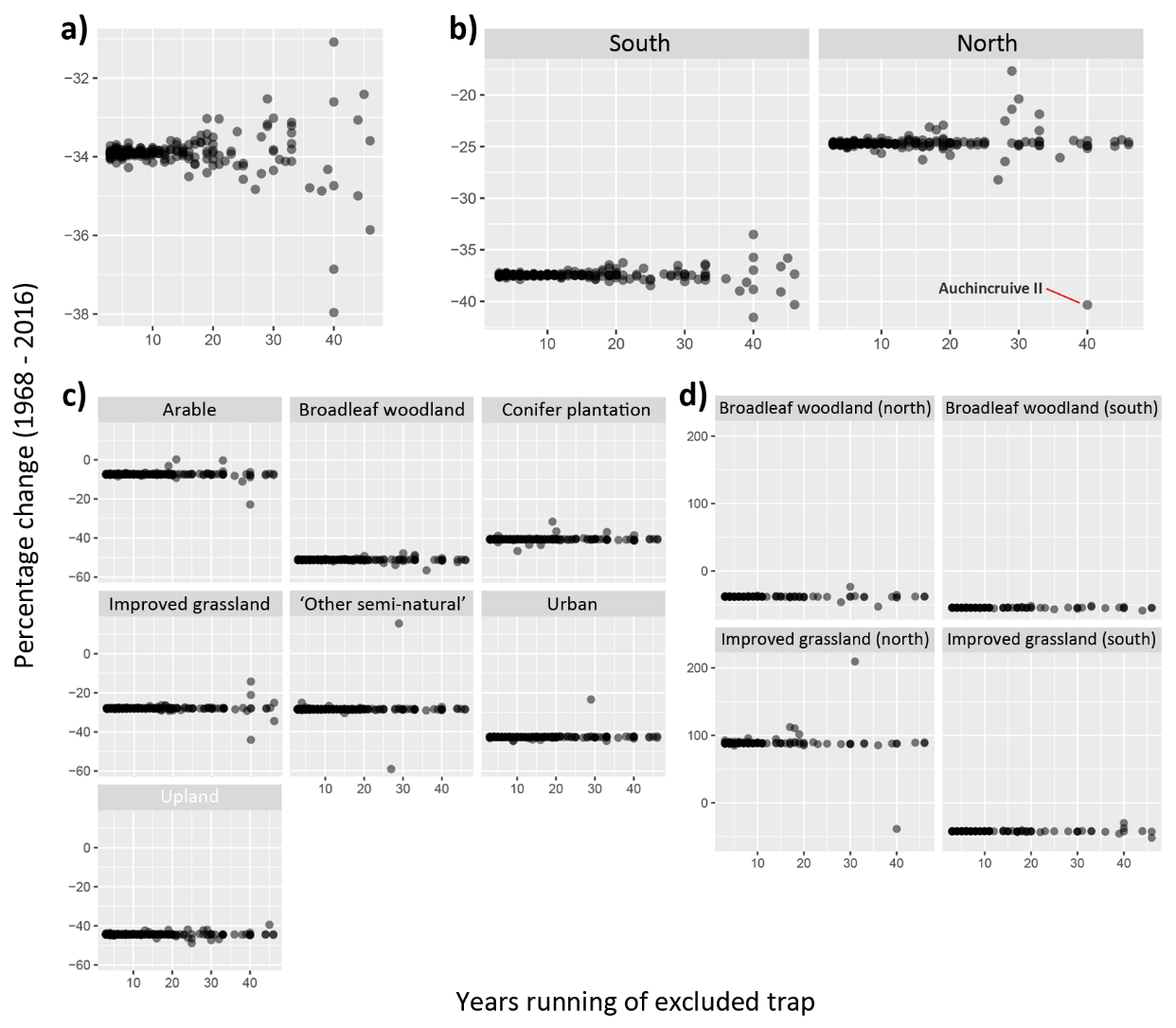


Fig S11. Results from jackknife sensitivity analysis. A GAMM was run in which one site was omitted at a time and the abundance trend 1968 – 2016 was recalculated. This was done for (a) all sites, (b) sites split by region, (c) sites split by habitat and (d) sites split by region and habitat (for broadleaf woodland and improved grassland only). Each point represents one model in which one site was removed. The x-axis shows the number of years the removed site was running for and the y-axis shows the estimated percent change with that site removed. The influential site Auchincruive II in the north is labelled.


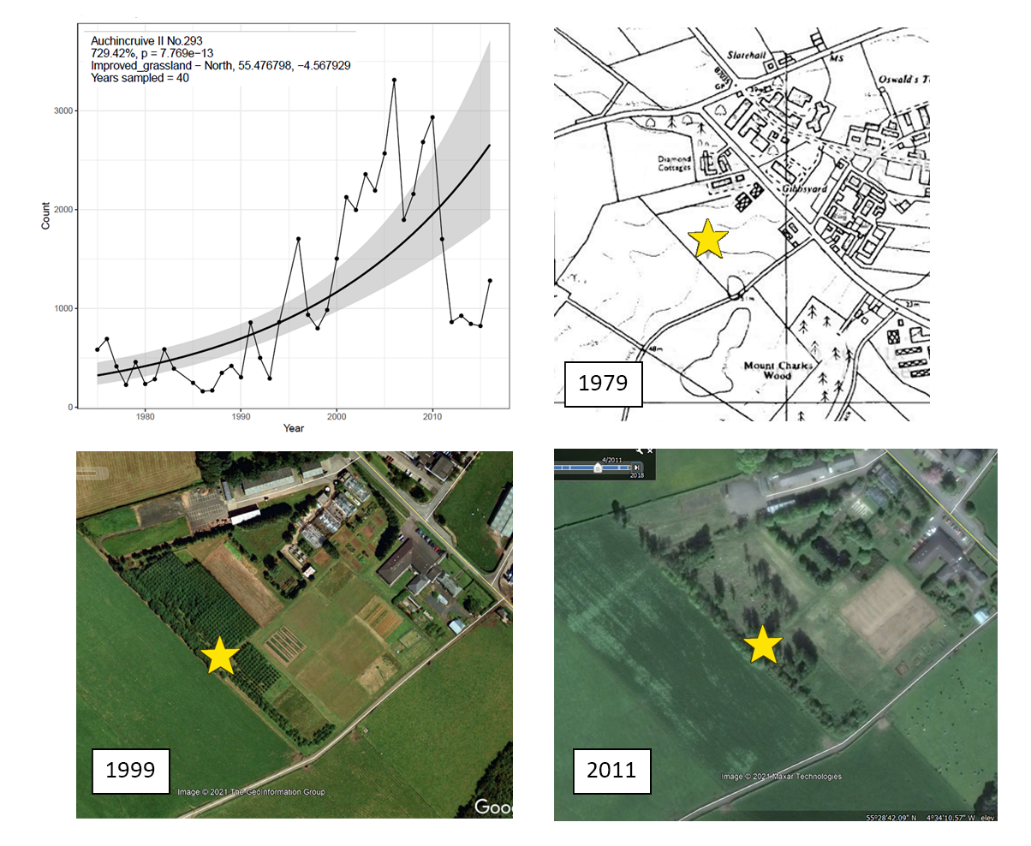


Fig S12. The site Auchincruive II over time – yellow star indicates the position of the trap. This site experienced a large increase in abundance of 729% over 40 years and has a large influence on the overall abundance trends for the north of the UK. The top left panel shows annual total counts 1975 – 2016 with model predictions from a linear model. The site was in an open arable field in 1979. The plantation visible in 1999 consists of poplars. In 2011, the poplar plantation has been cleared, with only some individual trees and shrubs left. It is likely that the increase in abundance followed the maturation of the dense stand of poplars around the site. A sudden drop in abundance is visible in 2010/11 when the plantation was cleared.


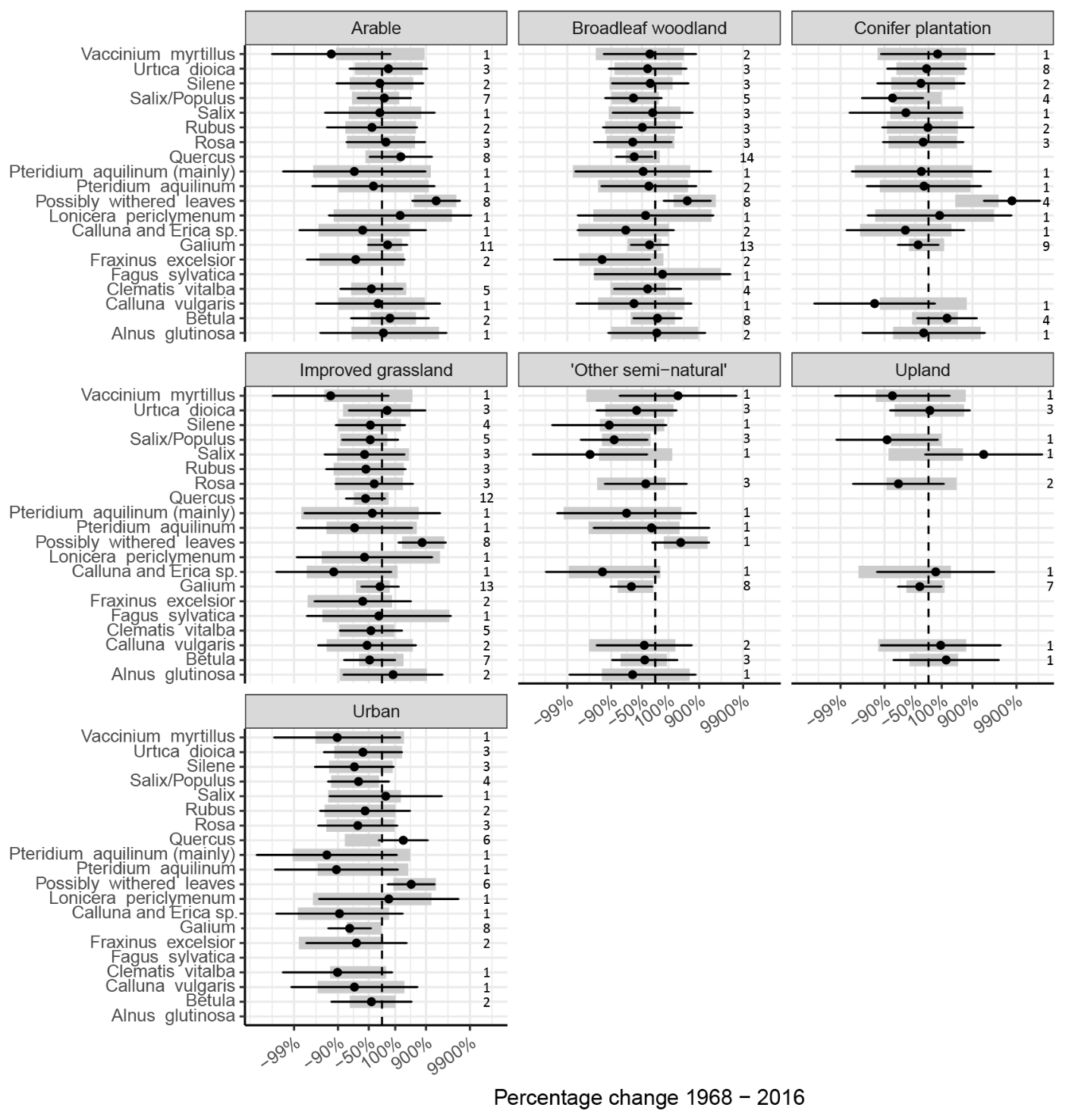


Fig S13. Percentage changes in moth abundance in species-specific models 1968 – 2016 for hostplant specialist species grouped by their hostplant in seven habitat types. Points and whiskers show estimated marginal means and 95% CIs from a LMM that specifies an interaction between hostplant and habitat. Grey bars show the 95% CIs from a model that specifies no interaction effect between hostplant and habitat. An LMM found that there was a significant interaction effect between habitat and hostplant (LRT, X^2^ = 181.4, p < 0.001) Dashed line shows zero percent change in each plot. The numbers down the right-hand side of each plot show the number of species included in each habitat-hostplant group.

# Reproducibility

All data and R script necessary to reproduce the analyses in this study, including figures, are provided in Data_and_analysis.zip.

# References

CONRAD, K. F., WARREN, M. S., FOX, R., PARSONS, M. S. & WOIWOD, I. P. 2006. Rapid declines of common, widespread British moths provide evidence of an insect biodiversity crisis. *Biological Conservation,* 132**,** 279-291.

DENNIS, E. B., FREEMAN, S. N., BRERETON, T. & ROY, D. B. 2013. Indexing butterfly abundance whilst accounting for missing counts and variability in seasonal pattern. *Methods in Ecology and Evolution,* 4**,** 637-645.

FOX, R., PARSONS, M., CHAPMAN, J., WOIWOD, I., WARREN, M. & BROOKS, D. 2013. The state of Britain’s larger moths 2013. *Butterfly Conservation and Rothamsted Research, Wareham, Dorset, UK,* 13.
